# Identification of an allatostatin C signaling system in mollusc *Aplysia*

**DOI:** 10.1038/s41598-022-05071-8

**Published:** 2022-01-24

**Authors:** Hui-Min Jiang, Zhe Yang, Ying-Yu Xue, Hui-Ying Wang, Shi-Qi Guo, Ju-Ping Xu, Ya-Dong Li, Ping Fu, Xue-Ying Ding, Ke Yu, Wei-Jia Liu, Guo Zhang, Jian Wang, Hai-Bo Zhou, Abraham J. Susswein, Jian Jing

**Affiliations:** 1 State Key Laboratory of Pharmaceutical Biotechnology, Institute for Brain Sciences, Chinese Academy of Medical Sciences Research Unit of Extracellular RNA, Jiangsu Engineering Research Center for MicroRNA Biology and Biotechnology, Advanced Institute for Life Sciences, Chemistry and Biomedicine Innovation Center, School of Life Sciences, Nanjing University, Nanjing, 210023 Jiangsu China; 2 School of Electronic Science and Engineering, Nanjing University, Nanjing, 210023 Jiangsu China; 3 Peng Cheng Laboratory, Shenzhen, 518000 China; 4 The Mina and Everard Goodman Faculty of Life Sciences, The Leslie and Susan Gonda (Goldschmied) Multidisciplinary Brain Research Center, Bar Ilan University, 52900 Ramat Gan, Israel; 5 Department of Neuroscience and Friedman Brain Institute, Icahn School of Medicine at Mount Sinai, New York, NY 10029 USA

**Keywords:** Cellular neuroscience, Feeding behaviour, Molecular neuroscience, Motor control

## Abstract

Neuropeptides, as pervasive intercellular signaling molecules in the CNS, modulate a variety of behavioral systems in both protostomes and deuterostomes. Allatostatins are neuropeptides in arthropods that inhibit the biosynthesis of juvenile hormones. Based on amino acid sequences, they are divided into three different types in arthropods: allatostatin A, allatostatin B, allatostatin C. Allatostatin C (AstC) was first isolated from *Manduca sexta*, and it has an important conserved feature of a disulfide bridge formed by two cysteine residues. Moreover, AstC appears to be the ortholog of mammalian somatostatin, and it has functions in common with somatostatin, such as modulating feeding behaviors. The AstC signaling system has been widely studied in arthropods, but minimally studied in molluscs. In this study, we seek to identify the AstC signaling system in the marine mollusc *Aplysia californica*. We cloned the AstC precursor from the cDNA of *Aplysia*. We predicted a 15-amino acid peptide with a disulfide bridge, i.e., AstC, using NeuroPred. We then cloned two putative allatostatin C-like receptors and through NCBI Conserved Domain Search we found that they belonged to the G protein-coupled receptor (GPCR) family. In addition, using an inositol monophosphate 1 (IP1) accumulation assay, we showed that *Aplysia* AstC could activate one of the putative receptors, i.e., the AstC-R, at the lowest EC_50_, and AstC without the disulfide bridge (AstC') activated AstC-R with the highest EC_50_. Moreover, four molluscan AstCs with variations of sequences from *Aplysia* AstC but with the disulfide bridge activated AstC-R at intermediate EC_50_. In summary, our successful identification of the *Aplysia* AstC precursor and its receptor (AstC-R) represents the first example in molluscs, and provides an important basis for further studies of the AstC signaling system in *Aplysia* and other molluscs.

## Introduction

Allatostatins are a group of neuropeptides in arthropods that are released in the retrocerebral corpora allata, and there inhibit the biosynthesis of juvenile hormones^1,2^. Indeed, they were initially discovered based on this bioactivity. Based on differences in their amino acid sequences, three types of allatostatins (allatostatin A, allatostatin B, allatostatin C) have been identified. Allatostatin C (AstC) was the last discovered of the three types of allatostatins. It was first identified in *Manduca sexta*^3^, and is characterized by 2 cysteines separated by 6 amino acids, whereas allatostatin A and B are amidated peptides without the two cysteines. The two cysteines in AstC likely form a disulfide bridge, which is presumably essential for activity. Allatostatin C genes also differ from allatostatin A or B in that they only encode a single C-type allatostatin peptide with the disulfide bridge^1^ whereas allatostatin A and B encode multiple functional peptides. Moreover, later work^4^ showed that, likely through gene duplications, there are two precursor genes for AstC in arthropods, with the second precursor producing a similar peptide, named AstCC. There is even evidence for a third precursor, AstCCC in some arthropods^5^. The receptors for AstC in arthropods have also been identified, with some species having two receptors^6,7^.

The AstC signaling system is of significant interest for two reasons. First, bioinformatic analyses^4,8–11^ have shown that allatostatin C appears to be a homolog of somatostatin (also with a disulfide bridge) in vertebrates^12–16^, indicating its importance across phyla. Second, bioinformatic analyses also suggest that AstC precursor and receptors are present in other invertebrates, particularly the superphylum lophotrochozoa (i.e., annelids, molluscs and brachiopods)^8–10^. Despite the bioinformatic evidence, however, to our knowledge, no AstC signaling system has been demonstrated in molluscs. We have examined allatostatin C function in an experimentally-advantageous system, the gastropod mollusc *Aplysia californica*. *Aplysia* has provided fundamental insight into the neural basis of motivated behaviors^17–33^, learning and memory^34–38^ and neuromodulation^39–42^, including neuropeptides^43–52^ and receptors^10,53,54^. In this work, we provide the first evidence for the presence of an AstC signaling system in molluscs. We took advantage of growing databases of the *Aplysia* genome and transcriptomes with increasing-quality sequence information that are becoming available (see Methods), and found candidate sequences of the precursor and two receptors for AstC. We cloned the precursor and the two receptors from *Aplysia* cDNA. We expressed the receptors in Chinese hamster ovary (CHO) cells, and used an IP1 accumulation assay to determine whether the peptides predicted from the precursor can activate the putative receptors. We found that the peptide with a disulfide bridge can indeed activate one of the two putative receptors, and the same peptide without the disulfide bridge activates the receptor with a significantly higher effective concentration. We also analyzed the evolutionary relationship between the *Aplysia* AstC precursor and receptors with those in other species, including somatostatin precursors and receptors in vertebrates. The study provides a basis for further investigations of AstC function in invertebrates other than arthropods, particularly in lophotrochozoa.

## Results

### Identifying a precursor for AstC and predicting peptides in *Aplysia*

**Figure 1:**
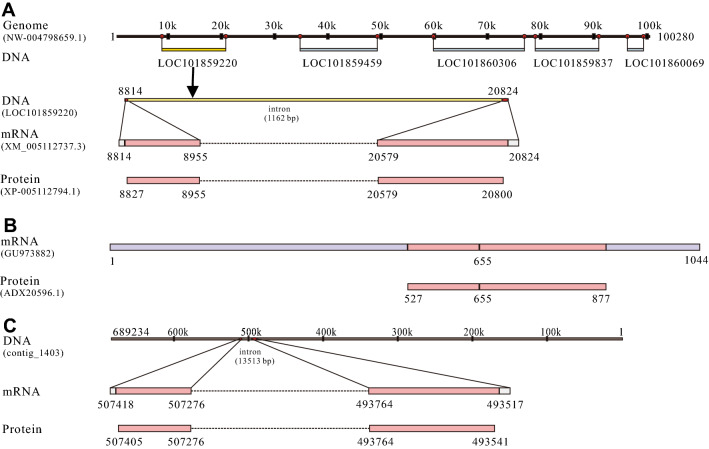
Gene expression mapping of the *Aplysia* Allatostatin C precursor. **(A)** A genome sequence from NCBI (NW-004798659.1) expresses 5 genes: LOC101859220; LOC101859459 (product: enolase); LOC101860306 (product: phosphatidylinositol); LOC101859837 (product: glyoxylate reductase/hydroxypyruvate reductase); LOC101860069 (product: glyoxylate reductase/hydroxypyruvate reductase). The first gene (LOC101859220) corresponds to a mRNA (XM_005112737.3, with an intron between 8955 to 20,579 bp), which produces an uncharacterized protein (XP-005112794.1). This protein is similar to Allatostatin C from other molluscs. **(B)** A mRNA from NCBI (GU973882), named *Aplysia californica* allatostatin C mRNA, complete CDS, was submitted by Moroz et al.^55^. It produces a protein, Allatostatin C (ADX20596.1), which is identical to XP-005112794.1 in **(A)**, but its untranslated regions at 5' and 3' ends are different from those of (XM_005112737.3) in **(A)**. **(C)** DNA contig-1403 (Note that the nucleotide number on top starts from the right) from *Aplysia* gene nucleotide databases (the AplysiaTools) expresses the same mRNA as in **(A)** (XM_005112737.3) and the protein generated from this mRNA is the same as those in **(A,B)**. Note that the numbers for the proteins refer to base pairs of corresponding mRNAs, not amino acids.

To identify putative precursor and receptors for *Aplysia* AstC, we began with a bioinformatic analysis. For the precursor, searching “*Aplysia* allatostatin C” in NCBI returned two entries: a predicted sequence (accession number: XM_005112737.3, which corresponds to a genome sequence: NW-004798659.1) (Fig. 1A), and an AstC precursor deposited in 2010 (mRNA accession number: GU973882) (Fig. 1B), which is likely based on an early large-scale sequencing project^55^. Using the RNA sequence from NCBI (XM_005112737.3), we also found an DNA sequence (DNA: contig_1403) in AplysiaTools (see Materials and Methods) that produces an identical mRNA sequence including the 5' and 3' ends of the noncoding regions as in mRNA (accession number: XM_005112737.3) (Fig. 1C). Note that the mRNA sequence (GU973882) produces an identical protein, but its noncoding regions are very different from the RNA sequence from NCBI (XM_005112737.3) and the AplysiaTools sequence, suggesting that the noncoding regions of the former sequence (GU973882) did not originate from the AstC precursor gene. Given that there are two precursor genes for AstC in arthropods^4^, with the second gene next to the first one, we examined whether there is a second mRNA following the AstC precursor that may encode an AstC peptide. In the genome scaffold (including NW-004798659.1 from NCBI and DNA contig-1403 from AplysiaTools that encode the AstC mRNA sequence in Fig. 1A,C), we found only one gene for the AstC related sequence, suggesting that it is likely that there is only a single AstC precursor in *Aplysia*.

**Figure 2:**
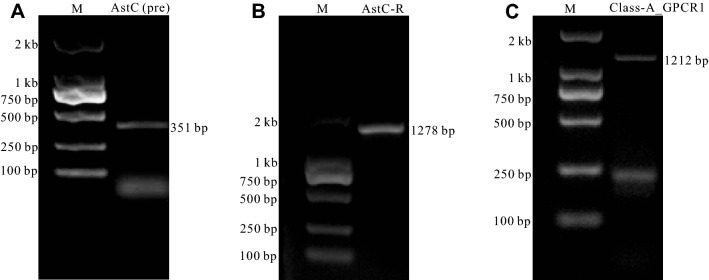
Cloning of *Aplysia* AstC precursor and putative receptors. **(A)** A PCR product for AstC precursor (AstC (pre)) gene with a length of 351 bp; **(B)** A PCR product for a putative receptor (AstC-R) with a length of 1278 bp; **(C)** A PCR product for a putative receptor (Class-A_GPCR1) with a length of 1212 bp. All the above mRNA sequences have been verified by sequencing. *M* marker.

After using bioinformatics to find a potential allatostatin C gene in *Aplysia*, it was important to identify the peptides that are generated by the precursor gene, and to find receptors that are responsive to the peptides. Here, we first designed primers (Supplementary Table 1) using the sequence we found, and performed PCR on cDNA of *Aplysia californica*, and obtained an mRNA of 351 bp in length (Fig. 2A, see Supplementary Fig. 1 for the complete gels). The gene sequence has been deposited into the NCBI database (GenBank accession number: OL546292). This mRNA sequence is similar to sequences from NCBI (i.e., XM_005112737.3 and GU973882) and AplysiaTools, and these sequences differ from each other in only one or two nucleotides (Supplementary Fig. 2). Moreover, these single nucleotide polymorphisms (SNPs) did not affect protein sequences, and the proteins from all four genes are identical. Thus, combined with earlier work, these data support the presence of an AstC precursor in *Aplysia*.

**Figure 3:**
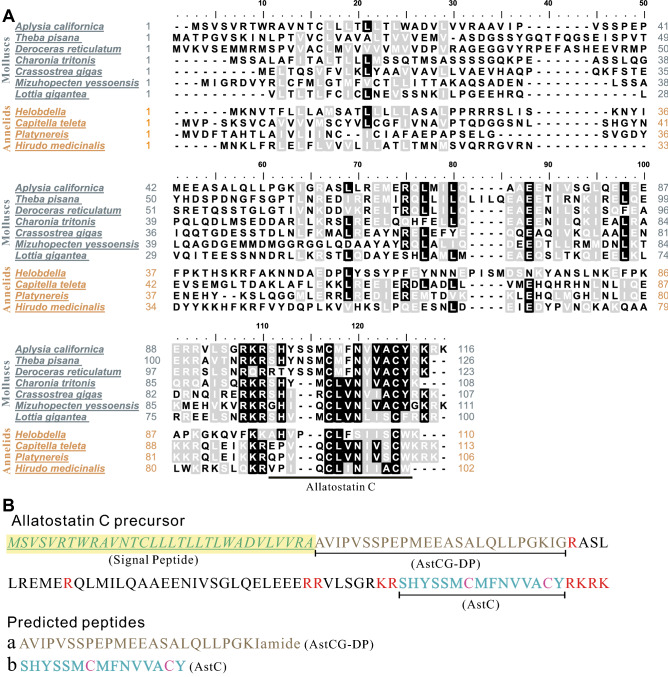
*Aplysia* AstC precursor vs. similar precursors in lophotrochozoa. **(A)** Comparison of *Aplysia* AstC precursors vs. similar precursors in lophotrochozoan (i.e., molluscs and annelids) using BioEdit v5.0.6 (ClustalW Multiple alignment—Graphic View). See Supplementary Table 2 for the source of the sequences used in this panel. Common names for each species: *Aplysia californica*: California sea hare, *Theba pisana*: white garden snail, *Deroceras reticulatum*: grey field slug, *Charonia tritonis*: giant triton snail, *Crassostrea gigas*: Pacific giant oyster, *Mizuhopecten yessoensis*: yesso scallop, *Lottia gigantea*: owl limpet*, Helobdella robusta*: Californian leech, *Capitella teleta*: bristle worm, *Platynereis dumerilii*: polychete ragworm, *Hirudo medicinalis*: medicinal leech. **(B)** The complete protein sequence of the *Aplysia* AstC precursor gene illustrating the signal peptide and two predicted peptides: AstC and AstCG-DP (G at the C-terminus is typically amidated as the final product). K, R, KR, RK (in red) are potential basic cleavage sites. Two cysteines are shown in purple, and likely form a disulfide bridge.

Next, we determined the identities and similarities between the *Aplysia* AstC precursor with AstC precursors from some selected invertebrate species and somatostatin precursors in some vertebrate species (Table 1, see Supplementary Table 2 for information on these sequences). Of the precursors examined, the *Aplysia* precursor is most closely related to molluscan AstC precursors, e.g., gastropods *Theba pisana* with a similarity of 53.1%, and *Deroceras reticulatum* with a similarity of 50.4%. Furthermore, we compared the 7 AstC precursors in molluscs and 4 in annelids (Fig. 3A, see Supplementary Table 2).

**Table 1:** Identities and similarities of AstC precursors from invertebrate species, and with somatostatins in vertebrates using BioEdit (pairwise alignment—calculate identity/similarity for two sequences).

Similarity matrix: BLOSUM62	AstC precursor (*Aplysia californica*)	
Name	Identities	Similarities
**Molluscs**		
AstC [*Theba pisana*]	32.03%	53.13%
AstC [*Deroceras reticulatum*]	40.00%	50.40%
AstC [*Charonia tritonis*]	28.57%	49.58%
AstC [*Crassostrea gigas*]	25.00%	41.38%
AstC [*Mizuhopecten yessoensis*]	23.28%	41.38%
AstC [*Lottia gigantea*]	24.14%	43.10%
**Annelids**		
AstC [*Helobdella robusta*]	13.45%	36.13%
AstC [*Capitella teleta*]	22.03%	38.14%
AstC [*Platynereis*]	18.97%	40.52%
AstC [*Hirudo medicinalis*]	18.10%	37.07%
**Arthropods**		
AstC [*Odontomachus brunneus*]	23.28%	40.52%
AstC [*Spodoptera frugiperda*]	21.26%	35.43%
AstC [*Mythimna unipuncta*]	20.47%	35.43%
AstC [*Drosophila melanogaster*]	19.69%	35.43%
AstC [*Scylla paramamosain*]	17.88%	34.44%
AstC [*Pseudaletia*]	17.74%	33.06%
AstC [*Bombyx mori*]	17.69%	33.85%
AstC [*Manduca sexta*]	16.54%	33.86%
AstC [*Samia ricini*]	15.87%	37.30%
AstC [*Anopheles gambiae*]	15.83%	30.00%
**Mammals**		
Somatostatin [*Mus pahari*]	11.67%	28.33%
Somatostatin [*Rattus norvegicus*]	11.67%	28.33%
Somatostatin [*Pan paniscus*]	11.67%	30.83%
Somatostatin [*Homo sapiens*]	11.67%	30.83%

**Figure 4:**
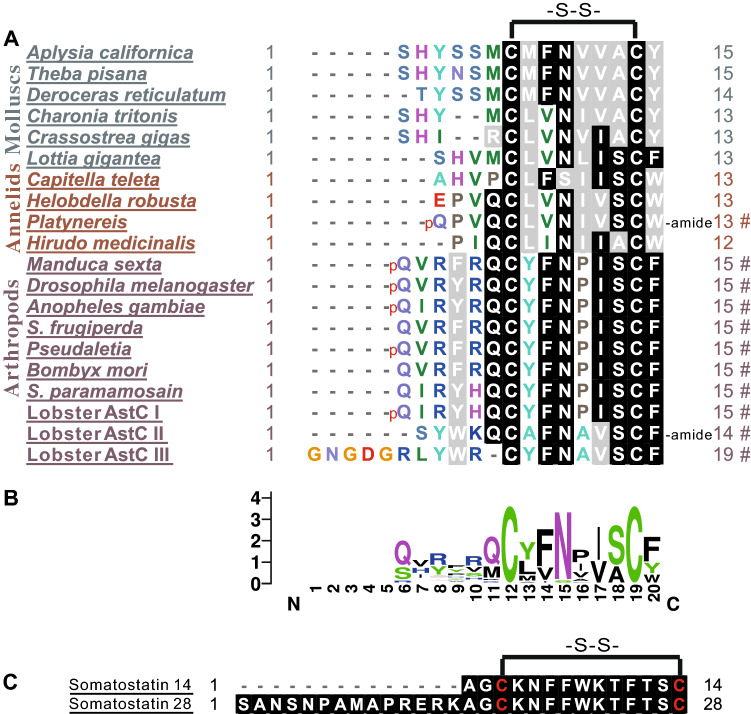
Comparison of AstCs from invertebrates, and with somatostatins in vertebrates. Comparison of selected AstC peptides from invertebrates using BioEdit (ClustalW Multiple alignment—Graphic View) **(A)** and a frequency plot for these sequences using Weblogo v2.8.2 (http://weblogo.berkeley.edu/logo.cgi) **(B)**. pQ indicates pyro-glutamic acid. At the right part of panel **A**, numbers denote the number of amino acids in a peptide, and # indicates that the sequence has been verified or studied before. See Supplementary Table 3 for the source of the sequences. **(C)** Somatostatin 14 and somatostatin 28 in mammals. Note that there are six amino acids between the two cysteines of allatostatin C in invertebrates, vs. ten amino acids between the two cysteines of somatostatins in mammals. *–S–S– *disulfide bridge.

Finally, we used NeuroPred^56^ to predict possible peptides that might be generated from the AstC precursor (Fig. 3B). They include at least two peptides: AstC: SHYSSMCMFNVVACY, and AstC gene-derived peptide (AstCG-DP): AVIPVSSPEPMEEASALQLLPGKIamide. Similar to AstC in other invertebrates, *Aplysia* AstC has two Cysteines with 6 amino acids in between. We also compared *Aplysia* AstC with AstCs in other invertebrate species and with somatostatin in mammals (Fig. 4, see Supplementary Table 3 for information on these sequences). Given that AstCs with the disulfide bridge in arthropods are bioactive, we hypothesized that *Aplysia* AstC with the disulfide bridge could activate an *Aplysia* AstC receptor (see Fig. 7).

### Identifying putative receptors for AstC in *Aplysia*

**Figure 5:**
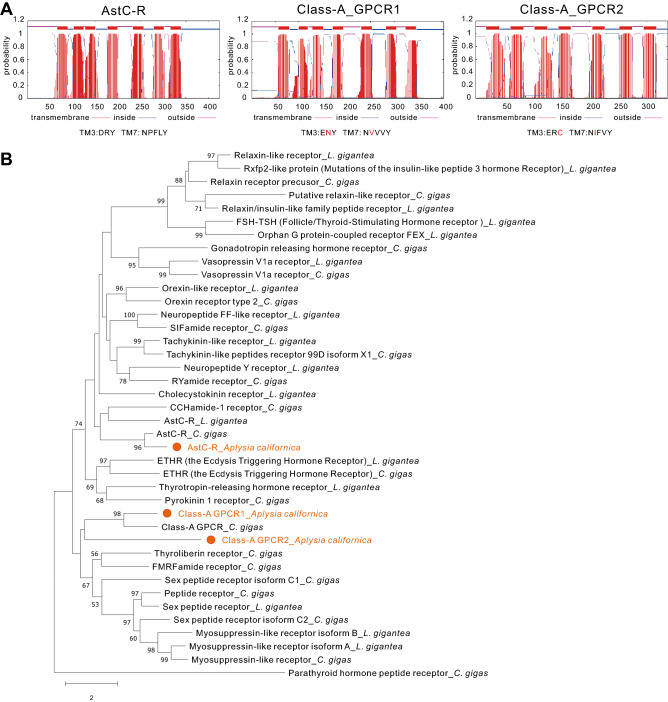
Bioinformatics of putative AstC receptors. **(A)** Prediction of 7TM of putative receptors: AstC-R, Class-A_GPCR1 and Class-A_GPCR2 using TMHMM. Conserved motifs in transmembrane domain 3 (TM3, D/ERY) and TM7 (NPXXY) are shown. The amino acids different from the motifs are shown in red. **(B)** A phylogenetic tree of three *Aplysia* proteins, AstC-R, Class-A_GPCR1, Class-A_GPCR2 (shown in orange) with *L. gigantea* and *C. gigas* Class A GPCR sequences from Jekely^8^ (see the Results and Supplementary Table 5) using MEGA X (See the bioinformatic section of the Methods for more details). Refer to the “Final name” worksheet in Supplementary Table 5 for the naming of the sequences. A Class B GPCR, Parathyroid hormone peptide receptor_*C. gigas*, was used as an outgroup. The tree suggests that AstC-R is an AstC receptor, whereas Class-A_GPCR1 and Class-A_GPCR2 are not. The tree is drawn to scale, with branch lengths measured in the number of substitutions per site. Numbers at the nodes are bootstrap values as percentage. Only bootstrap values greater than 50 are shown.

To identify putative receptors, we searched “*Aplysia* allatostatin C receptor” in NCBI, but this search did not return any sequences. Because of the homology of allatostatin C and somatostatin^4,8,9,11^, we then tried to search “*Aplysia* somatostatin receptor” in NCBI, which did return seven sequences (Supplementary Table 4). In Supplementary Table 4, we also indicated whether these seven sequences are present in AplysiaTools. Next, we used NCBI Conserved Domain Search and TMHMM sever 2.0 to predict whether these seven sequences are GPCRs. Among them, three are predicted to have 7 transmembrane domains (Fig. 5A), which presumably are complete GPCR sequences. These three sequences are also present in AplysiaTools databases (see Supplementary Table 4).

To determine whether the three putative GPCRs might be related to AstC receptors, we blasted each sequence in NCBI in species where more protein sequences have been studied, i.e., *Caenorhabditis elegans*, *Drosophila melanogaster*, *Danio rerio* and *Mus musculus*. For the protein with accession number: XP_005095139.1 (mRNA: XM_005095082.3), named somatostatin receptor type 2-like receptor in NCBI, a number of sequences named somatostatin receptors or AstC receptors with low E-values came up at these searches in several invertebrate and vertebrate species (Supplementary Table 5), suggesting that this protein might be related to AstC receptors. We therefore tentatively named it as AstC-R. For the protein with accession number: XP_005096107.1 (mRNA: XM_005096050.3), named somatostatin receptor type 3-like receptor in NCBI, and the protein with accession number: XP_012945941.1 (mRNA: XM_013090487.2), also named somatostatin receptor type 2-like receptor in NCBI, these searches did not return useful known proteins with low E-values (< 1E-4) and high query coverage (> 50%) (Supplementary Table 5). Therefore, we used Pfam (http://pfam.xfam.org/search#tabview=tab1) to blast both proteins, and both are classified as Class A GPCRs (rhodopsin family). Thus, we tentatively named the first protein as Class-A_GPCR1, and the second one as Class-A_GPCR2 (Supplementary Table 4 and 5).

**Figure 6:**
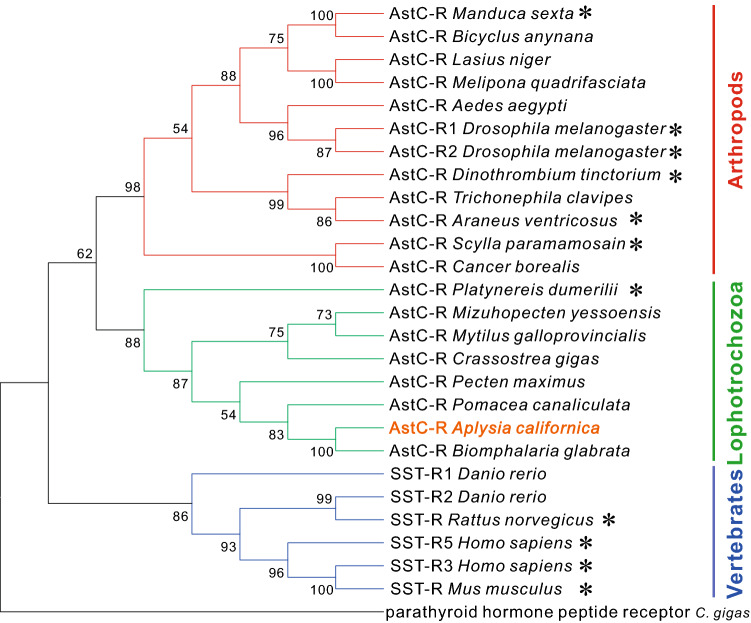
A phylogenetic tree of AstC-R in *Aplysia* with predicted or verified AstC receptors in invertebrates and somatostatin receptors (SST-Rs) in vertebrates. The tree was generated using MEGA X with 1000 replicates (See the bioinformatic section in Methods for more details and Supplementary Table 6 for information on the sequences). This phylogenetic tree indicates that *Aplysia* AstC-R is more closely related to a sequence in mollusc *Biomphalaria*. * indicates that the receptor has been studied/verified. “Parathyroid hormone receptor 1 *C. gigas*” is a Class B GPCR used as an outgroup (see Supplementary Table 5). Numbers at the nodes are bootstrap values as percentage. Only bootstrap values greater than 50 are shown.

To obtain a better view of phylogenetic relationship between the three proteins, we decided to construct a phylogenetic tree with a number of Class A GPCRs in *Lottia giagantea* and *Crassostrea gigas* from the Supplementary File, sd02.rtf, of Jekely 2013^8^ and two other sequences of *Crassostrea gigas* (Cg_XP_011450519.2, Cg_XP_011428314.2). We selected protein sequences with more than 340 amino acids, and eliminated duplicated sequences from file “sd02.rtf”. As described in the last paragraph, we blasted these sequences in *Caenorhabditis elegans*, *Drosophila melanogaster*, *Danio rerio* and *Mus musculus* and annotated them (Supplementary Table 5, except Cg_XP_011450519.2, which did not return a proper name, so we named it “Class-A_GPCR *C gigas*” based on Pfam). Then, we added the three *Aplysia* sequences, together with AstC-R sequence of *Crassostrea gigas* (see also Supplementary Table 5), and re-ran the phylogenetic tree (Fig. 5B). The tree showed that *Aplysia* AstC-R clustered together with *Crassostrea gigas* AstC-R, supporting the hypothesis that *Aplysia* AstC-R might be an AstC receptor, whereas the two other *Aplysia* proteins, Class-A_GPCR1 and Class-A_GPCR2, are not closely related to AstC receptors. Finally, we generated a phylogenetic tree of *Aplysia* AstC-R with AstC-Rs from selected species in arthropods, lophotrochozoa and some somatostatin receptors in vertebrates (Fig. 6, see Supplementary Table 6 for information on these sequences). We also compared most of the putative molluscan AstC-Rs with *Aplysia* AstC-R using BioEdit (Supplementary Fig. 3, see Supplementary Table 6). The data indicate that of the sequences examined, *Aplysia* AstC-R is most closely related to the sequence from pulmonate gastropod, *Biomphalaria glabrata* (similarity: 70.98%). In addition, AstC-R appears to be more similar to AstC-R1 (similarity: 41.71%) than to AstC-R2 (similarity: 40.6%) in *Drosophila* (see the clustal table in Supplementary Table 6).

We chose to pursue the research by first cloning *Aplysia* AstC-R. We also cloned *Aplysia* Class-A_GPCR1, which was to be used as a control. We designed primers (Supplementary Table 1) using the two sequences, and successfully cloned mRNAs for both AstC-R and Class-A_GPCR1 (Fig. 2B, C, see Supplementary Fig. 1 for the complete gels). The gene sequences have been deposited into the NCBI database (AstC-R GenBank accession number: OL546293; Class-A_GPCR1 GenBank accession number: OL546294). To search for other sequences that might be related to AstC-R, we used the cloned AstC-R sequence to blast both the transcriptome and the genome of the AplysiaTools databases, but we did not find any additional related sequences.

### Activation of putative receptors by AstC peptides

**Figure 7:**
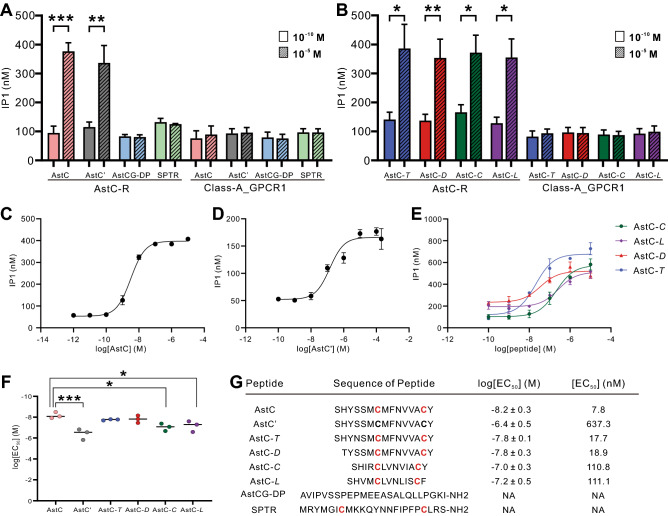
Activation of *Aplysia* AstC and related peptides on putative receptors determined using IP1 accumulation assay. **(A,B)** Screening of potential activation of peptide ligands on putative receptors (AstC-R and Class-A_GPCR1) using two concentration: 10^–10^ M and 10^–5^ M. At 10^–10^ M, a peptide activated a receptor minimally, if at all, so it is used as a control. **(A)** AstC, AstC' (AstC without the disulfide bridge), AstCG-DP, *Aplysia* SPTR (apSPTR-GP-DP2). **(B)** AstCs from four other molluscan species: AstC-*T* (*Theba pisana*), AstC-*D* (*Deroceras reticulatum*), AstC-*C* (*Crassostrea gigas*) and AstC-*L* (*Lottia giagantea*). AstC, AstC-*T*, AstC-*D*, AstC-*C* and AstC-*L* on AstC-R: n = 6, AstC' on AstC-R: n = 5; n = 3 for all other tests. AstC and AstC' significantly increased IP1 concentration when acting on AstC-R, suggesting that AstC and AstC' are ligands for AstC-R. AstC-*T*, AstC-*D*, AstC-*C* and AstC-*L* also significantly increased IP1 concentration when acting on AstC-R. In contrast, AstCG-DP and SPTR did not activate AstC-R significantly. Moreover, Class-A_GPCR1 did not respond significantly to any of the peptides. Paired t-test, *, P < 0.05, **, P < 0.01, *** P < 0.001, error bar: SEM. **(C–E)** Representative examples of dose response curves of the activation of AstC-R by *Aplysia* AstC **(C)**, AstC' **(D)**, and AstC-*T*, AstC-*D*, AstC-*C* and AstC-*L* **(E)**. Each data point is from two wells. Error bars, SEM. **(F)** Comparison of log[EC_50_] from the six peptides (n = 3 for each) shown in **(C–E)**. One-way ANOVA, F(5, 12) = 8.47, P < 0.01. Bonferroni post-hoc test: *, P < 0.05, ***, P < 0.001. **(G)** Sequences of all peptides tested and summary of the average log[EC_50_] and EC_50_ on AstC-R. Cysteines in red denote the disulfide bridge in the peptide.

We cloned AstC-R and Class-A_GPCR1 into pcDNA3.1 plasmids, and expressed them in CHO cells. We then used the IP1 accumulation assay that detects IP1 generated in the Gαq pathway (see Methods) to determine whether our predicted *Aplysia* peptides (i.e., AstC or AstCG-DP) and other related peptides which we synthesized (see Fig. 7G) could activate the receptors. In preliminary experiments, we only transfected plasmids for a putative receptor in CHO cells. However, none of the receptors responded to AstC or AstCG-DP, suggesting that it is possible that these receptors did not associate with the native Gαq in CHO cells. Thus, for all the experiments shown in Fig. 7, we co-transfected plasmids for the putative receptor and Gα16 plasmids in CHO cells. Because Gα16 is a promiscuous Gαq protein that will bind to most GPCRs^10,53^, this procedure would ensure an IP1 response when a potential ligand binds to its GPCR.

We first screened IP1 responses of the two peptides, AstC and AstCG-DP, at two concentrations (10^–10^ M and 10^–5^ M) on the two receptors: AstC-R and Class-A_GPCR1 (Fig. 7A). At 10^–10^ M, a peptide activated a receptor minimally, if at all, so it is used as a control. We also tested the effects of several other peptides, including AstC without the disulfide bridge (named AstC'), *Aplysia* SPTR (apSPTR-GP-DP2), another *Aplysia* peptide also with a disulfide bridge^52^. In addition, we selected several AstCs predicted from four other molluscan species (Fig. 4A), i.e., AstC-*T* (*Theba pisana*), AstC-*D* (*Deroceras reticulatum*), AstC-*C* (*Crassostrea gigas*) and AstC-*L* (*Lottia giagantea*) (Fig. 7G). We found that AstC-R was responsive to AstC and AstC', but not to AstCG-DP or SPTR (Fig. 7A). The data suggest that AstC and AstC' were the active peptides, and AstC-R is their receptor. Moreover, *Aplysia* AstC-R responded to all AstCs from the four other molluscan species (Fig. 7B). On the other hand, Class-A_GPCR1 did not respond significantly to any of the peptides tested (Fig. 7A,B), supporting the bioinformatic analysis showing that Class-A_GPCR1 is not an AstC receptor.

Furthermore, for the six peptides that had a significant effect on AstC-R in the initial screening (Fig. 7A,B), we used multiple concentrations of the peptides, ranging from 10^–12^ M to 10^–4^ M to determine the dose response curve of peptide activation on the AstC-R (Fig. 7C–E). We found that log[EC_50_] of AstC is the lowest, −8.2 ± 0.3 M (n = 3), whereas that of AstC' is the highest, −6.4 ± 0.5 M, which was significantly higher than that of AstC (Fig. 7F,G), indicating that the disulfide bridge plays an important role in the activity of the receptor. The peptides with the disulfide bridge but having different variations of amino acid sequences from four other molluscan species had log[EC_50_] at intermediate values (Fig. 7F,G), indicating that both the disulfide bridge and amino acid sequences play some roles in the receptor activity.

## Discussion

Growing databases of the *Aplysia* genome and transcriptome are becoming available, but relatively few studies have taken advantage of this information (see^53,54^). Here, we have used bioinformatics, molecular biology, and a cell-based assay to demonstrate for the first time that an allatostatin C signaling system is present in the mollusc *Aplysia*. This appears to be the first example of a clear demonstration of an AstC signaling system in molluscs. Previous bioinformatic studies in several molluscs predicted the presence of an allatostatin C precursor (Fig. 3, e.g.,^57^) and of AstC with the disulfide bridge (Fig. 4) as well as the receptors (Fig. 6, e.g.,^58,59^). However, these studies did not report activation of any of the putative AstC receptors. In contrast, there is a recent work that identified allatostatin C and its putative receptor in an annelid, *Platynereis dumerilii*^10^. However, the EC_50_ of this *Platynereis* receptor was quite high (1000 or 1200 nM) compared with the EC_50_ of *Aplysia* AstC-R (7.8 nM). Thus, it is possible that there is another AstC receptor in *Platynereis* that could respond to AstC with a lower EC_50_. Annelids are related to molluscs in that they are both within the superphylum: lophotrochozoa.

In this age of genomics, a large amount of sequence information that is available has facilitated the analysis of evolutionary relationships of neuropeptide signaling systems across phyla^4,8–11^, which has in turn predicted the presence of diverse peptide signaling systems in specific species. However, the ultimate evidence for the presence of any particular signaling system in a species needs to be obtained using molecular biology, cell-based assays and other approaches that conclusively prove such a prediction to be correct. In the present work, we have successfully identified one precursor and one receptor for AstC (Figs. 2A,B, 7) in *Aplysia*, although we cannot exclude the possibility as yet that there may be one more precursor or one more receptor, as in some arthropods. More work is needed to resolve these issues. Notably, we demonstrated the important role of the disulfide bridge in AstC because compared with the AstC with the disulfide bridge, the AstC without the disulfide bridge had a significantly higher log[EC_50_] on AstC-R (Fig. 7F). In addition, the amino acid sequence of *Aplysia* AstC is also important for receptor activity because four molluscan AstCs with the disulfide bridge had intermediate log[EC_50_] and the four sequences vary somewhat from *Aplysia* AstC, whereas *Aplysia* SPTR with a disulfide bridge is inactive apparently due to the fact that its sequence is very different from *Aplysia* AstC (Fig. 7).

Our analyses indicate that the protein sequence encoded by the AstC precursor we identified is identical to ones available in current databases (Fig. 1 and Supplementary Fig. 2), suggesting that sequences in the available public databases for *Aplysia* are credible and useful in predicting the precursor gene and putative peptides. In contrast, the receptors we identified required much more work to determine their sequences and whether they actually function as a receptor for AstC. Indeed, the significance of the present work can be appreciated by the fact that searching *Aplysia* somatostatin receptors have returned up to seven predicted sequences in NCBI, but only three of them are complete sequences, and among them, only one is significantly activated by *Aplysia* AstC. Thus, bioinformatics and experimental work are both necessary for identification of a peptide signaling system in a particular species. This is also a cautionary tale for evolutionary analysis of peptide precursors and especially receptors based only on bioinformatics.

Overall, our study is an important initial advance toward studying allatostatin C signaling system in *Aplysia*. Future work will use mass spectrometry (i.e., to demonstrate the specific form of AstC expressed in the *Aplysia* CNS), in situ hybridization, physiological techniques to provide further proof of a functional AstC signaling system in *Aplysia*, and to demonstrate its specific physiological roles. Indeed, our work provides an important basis for the study of functional roles of AstC signaling system invertebrates, other than arthropods. Although allatostatin C was initially found based on its bioassay to inhibit the biosynthesis of juvenile hormones in retrocerebral corpora allata in insects^3^, growing evidence suggests that it has multiple functions (see^60^) as do many peptides^41,61,62^. For example, both allatostatin C^60^ and somatostatin^14,15^ may play a modulatory role in feeding. In addition, a recent work has shown that the AstC signaling system plays an important role in modulating circadian activity in *Drosophila*^63^, supporting diverse roles of AstC. Invertebrates other than arthropods, such as *Aplysia*, do not have corpora allata or juvenile hormones. Thus, it would be of great interest to determine whether *Aplysia* AstC plays an important role in modulating motivated behaviors such as feeding and locomotion. Because feeding and locomotor networks are well studied in *Aplysia*^17–19,21,23–25,29–33,51,52,64–68^, we expect that future studies will provide novel understanding of synaptic and circuit roles of the AstC signaling system in various behavioral networks. Finally, studying the AstC signaling system in molluscs will inform us how the genes and functions of the AstC and somatostatin signaling systems may have evolved across phyla.

## Material and methods

### Subjects and reagents

Experiments were performed on *Aplysia californica* (100–350 g) obtained from Marinus, California, USA. *Aplysia* are hermaphroditic (i.e., each animal has reproductive organs normally associated with both male and female sexes). Animals were maintained in circulating artificial seawater at 14–16 °C and the animal room was equipped with a 24 h light cycle with light period from 6:00 am to 6:00 pm. All chemicals were purchased from Sigma-Aldrich unless otherwise stated.

### Bioinformatic analysis of peptide precursors and receptors

We first used NCBI to search specific sequences of interests. In addition, we also searched AplysiaTools databases (Dr. Thomas Abrams, University of Maryland, USA) to obtain additional sequences for comparison. These latter databases (http://aplysiatools.org) include databases for *Aplysia* transcriptome and *Aplysia* genome.

The open reading frames (ORFs) of the AstC precursor, putative receptor full-length cDNA sequences were obtained using ORF Finder (https://www.ncbi.nlm.nih.gov/orffinder/). For the AstC precursor, the putative signal peptide was predicted using SignalP-5.0 (http://www.cbs.dtu.dk/services/SignalP/) and the putative peptides encoded by the AstC precursor were predicted using NeuroPred (http://stagbeetle.animal.uiuc.edu/cgi-bin/neuropred.py). We also compared the AstC precursor and neuropeptide sequences with those of other species using BioEdit software and generated a frequency plot of each amino acid (aligned from c-terminus) using a Weblogo software (http://weblogo.berkeley.edu/logo.cgi). For the putative AstC receptors, transmembrane domains were predicted using TMHMM Server v. 2.0 (http://www.cbs.dtu.dk/services/TMHMM/). For proteins that were difficult to annotate using blast, we also used Pfam database (http://pfam.xfam.org/search#tabview=tab1) to determine what type of a protein it is. The phylogenetic trees of sequences from different species were constructed by MEGA X software (https://www.megasoftware.net/) using alignment by MUSCLE and the maximum likelihood method with 1000 replicates. For Fig. 5B, we used LG + G + F model to generate our final tree; for Fig. 6, JTT model was performed which was different from Fig. 5B. The selection of the models was based on the results of MEGA analysis.

### Cloning of mRNA in *Aplysia*

#### RNA extraction

After anesthesia with 30–50% of the body weight with 333 mM MgCl_2_, *Aplysia* cerebral, pleural-pedal, buccal and abdominal ganglia were dissected out and maintained in artificial seawater containing the following (in mM): 460 NaCl, 10 KCl, 55 MgCl_2_, 11 CaCl_2_, and 10 HEPES buffer, pH 7.6, in a dish lined with Sylgard (Dow Corning). RNA was prepared from the *Aplysia* ganglia using the TRIzol reagent method. Specifically, the dissected ganglia were placed into 200 μl TRIzol (Sigma, T9424) and stored at -80 °C until use. The frozen ganglia in TRIzol were thawed and homogenized with a plastic pestle, then TRIzol was added to a total volume of 1 ml, which were incubated at room temperature for 10 min. Then, 200 μl chloroform was added, and the solution was mixed thoroughly by shaking, and let stand on ice for 15 min. The solution was centrifuged (12,000 × *g*, 4 °C, 15 min), and the supernatant was added to an equal volume of isopropanol. The tube was shaken gently by hand and let stand at -20 °C for 2 h. After 2 h, it was centrifuged (12,000 × *g*, 4 °C, 15 min) again, the supernatant was discarded, 1 ml of 75% ethanol/water was added, and the centrifuge tube was shaken gently by hand to suspend the pellet. It was centrifuged (12,000 × *g*, 4 °C, 10 min), the supernatant discarded and the precipitant was dried at room temperature for 5–10 min. Finally, 30 μl of nuclease-free water was added to dissolve the RNA pellet, and the RNA concentration was determined with a Nanodrop ND-1000 spectrophotometer (Thermo Fisher Scientific).

#### Reverse transcription

Using the above extracted RNA as a template, cDNA was synthesized by reverse transcription using PrimeScript RT Master Mix Kit (Takara, RR036A) according to the instructions and then stored at − 20 °C until use. The synthesized first-strand cDNA serves as a template for PCR.

#### PCR

The synthesized cDNA above was used as a template for PCR. Each pair of specific primers was designed (Supplementary Table 1) in Primer Premier 6 and Oligo7, based on protein coding sequences for the AstC precursor and putative receptors. The PCR reaction was performed with 98 °C/2 min pre-denaturing, 98 °C/10 s denaturing, ~ 64 °C (depending on the specific primers: see Supplementary Table 1)/15 s annealing, 72 °C/30 s extension and 72 °C /5 min re-extension for 35 cycles. The PCR products were subcloned into vector pcDNA3.1( +) and sequenced to ensure the sequence was correct.

### IP1 accumulation assay

Inositol monophosphate 1 (IP1) accumulation assay measures concentration of IP1, that is hydrolyzed from the second messenger, inositol monophosphate 3 (IP3), generated by Gαq pathway when a G-protein coupled receptor (GPCR) expressed in CHO-K1 cells is activated by an appropriate ligand. In order to express the *Aplysia* putative receptors transiently in CHO-K1, the cDNA was cloned into the mammalian expression vector pcDNA3.1( +). CHO-K1 cells (Procell, CL-0062) were cultured in F-12K medium (Gibco, 21127-022) with 10% fetal bovine serum (Genial, G11-70500) at 37 °C in 5% CO_2_. Transfection experiments were performed when the cells were grown to 70–90% confluence. In preliminary experiments, for each dish (60-mm diameter), 4 μg of the putative receptor plasmids [in pcDNA3.1( +)] were mixed with 400 μl of Opti-MEM (Gibco, 11058021), followed by the addition of 15 μl of Turbofect (Thermo Fisher Scientific, R0531). However, none of the receptors responded to AstC or AstC gene-derived peptide (AstCG-DP, see Fig. 3B and Results), suggesting that these receptors may not associate with the native Gαq in CHO cells. Thus, for all experiments shown in Fig. 7, 3 μg of the putative receptor plasmids [in pcDNA3.1( +)] and 3 μg of Gα16 plasmids [in pcDNA3.1( +)] were co-transfected in the above procedure to ensure that the ligand-receptor binding would generate a IP1 response because Gα16 is a promiscuous Gαq protein that will bind to most GPCRs^10,53^. The CHO cells with the reagents added above were mixed gently, and incubated at room temperature for 15 min. The DNA/Turbofect mixture dropwise was then added to the dish, and the cells were incubated at 37 °C in 5% CO_2_ overnight. The next day, the cells were trypsinized and reseeded in opaque white 96-well half-area (Corning, 3688) or 384-well tissue culture-treated plates (Corning, 3570) at a density of 20,000 cells/well in F-12K and 10% FBS and incubated at 37 °C in 5% CO_2_ overnight. On the third day, the activation of the putative receptor was detected by monitoring IP1 accumulation using IP1 detection kit (Cisbio, 62IPAPEB) in Tecan Spark. Except for using 0.5 × reagent, all other procedures were performed in accordance with the IP1 detection kit manufacturer’s instructions. Peptides are synthesized by Sangon Biotech, Guoping Pharmaceutical or ChinaPeptides (Supplementary Fig. 4), and are aliquoted in 50 nmol EP tubes, stored at −20 °C until use.

## Supplementary Information

Supplementary Figures.

Supplementary Figure 4.

Supplementary Table 1.

Supplementary Table 2.

Supplementary Table 3.

Supplementary Table 4.

Supplementary Table 5.

Supplementary Table 6.
