# Supplementary material for: Identification of an allatostatin C signaling system in mollusc Aplysia
Source: Sci Rep. 2022 Jan 24;12:1213. doi: 10.1038/s41598-022-05071-8 (PMC8786951; doi:10.1038/s41598-022-05071-8)
Supplement: Supplementary file 1 — Supplementary Figures. [file 41598_2022_5071_MOESM1_ESM.docx]

**Scientific Reports**

Supplement to: **Identification of an allatostatin C signaling system in mollusc *Aplysia***

Hui-Min Jiang^1,6^, Zhe Yang^1,6^, Ying-Yu Xue^1^, Hui-Ying Wang^1^, Shi-Qi Guo^1^, Ju-Ping Xu^1^, Ya-Dong Li^1^, Ping Fu^1^, Xue-Ying Ding^1^, Ke Yu^1^, Wei-Jia Liu^1^, Guo Zhang^1*^, Jian Wang^2,3*^, Hai-Bo Zhou^2,3*^, Abraham J. Susswein^4^, Jian Jing^1,3,5*^

Supplementary information includes 6 supplementary tables (as separate Excel files), and 4 supplemental figures (3 in this file, Supplementary Fig. 4 as a separate PDF file).


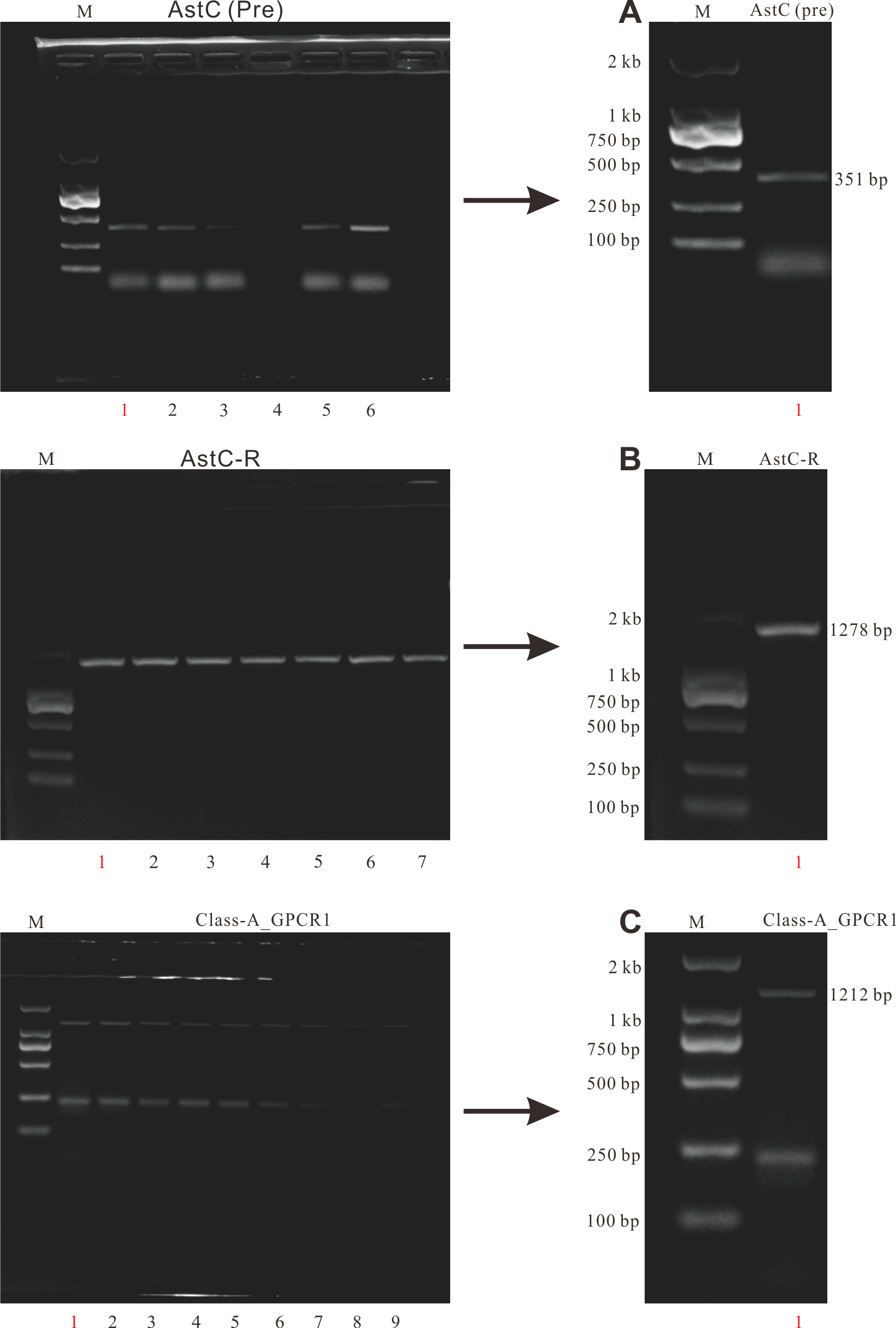


**Supplementary Figure 1. Complete gels for PCR experiments**. Left panels: complete gels; Right panels, corresponding cropped gels shown in Fig. 2. Lane 1 in all panels is shown in Fig. 2. M: Marker.


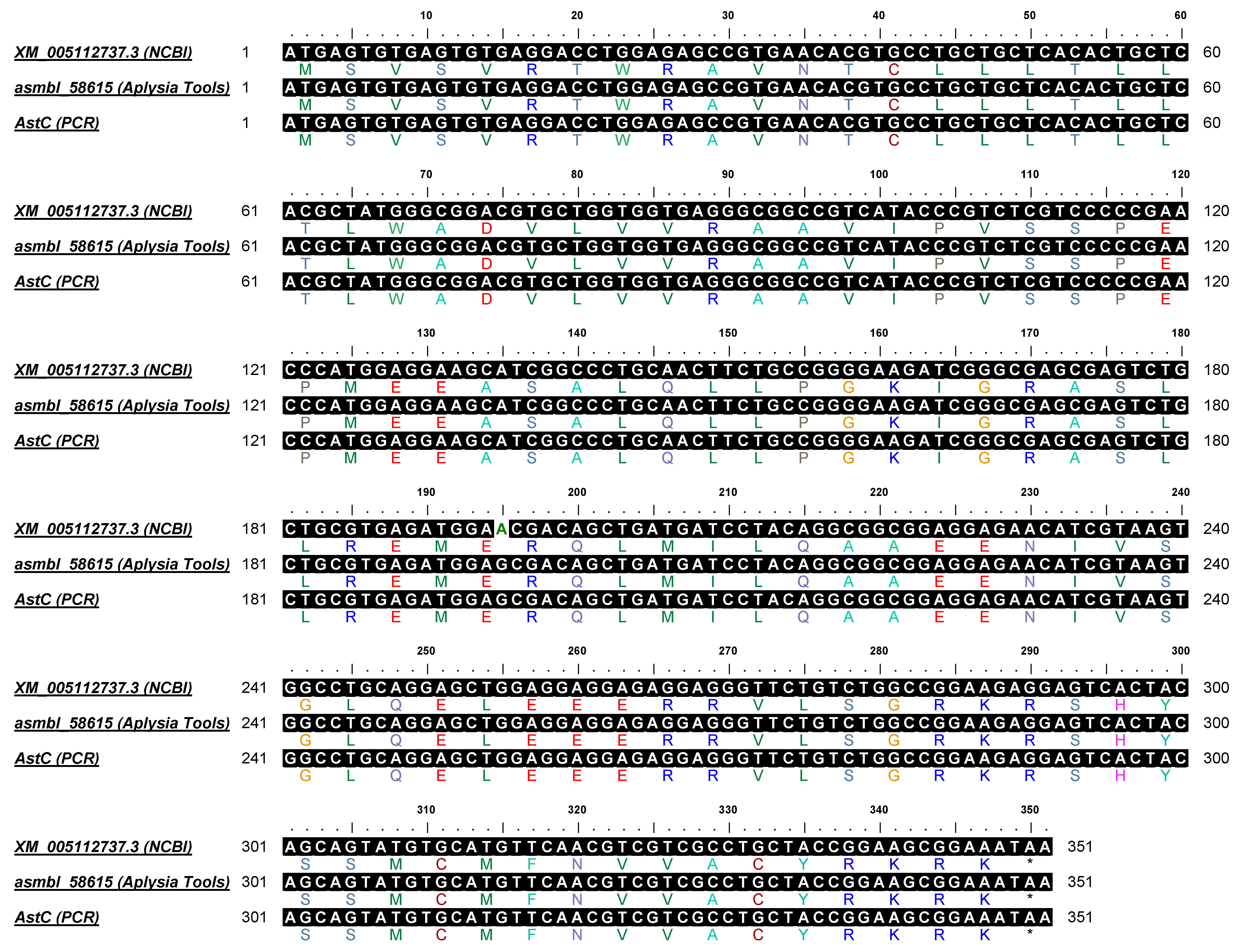


**Supplementary Figure 2. Comparison of CDS sequences of the *Aplysia* AstC precursor from NCBI, AplysiaTools, and the cloned sequence (see Fig. 2A).** The alignment is performed using BioEdit v5.0.6 (ClustalW Multiple alignment - Graphic View). There are one or two nucleotide differences between the mRNA sequences, but all mRNAs generate the same protein sequence.


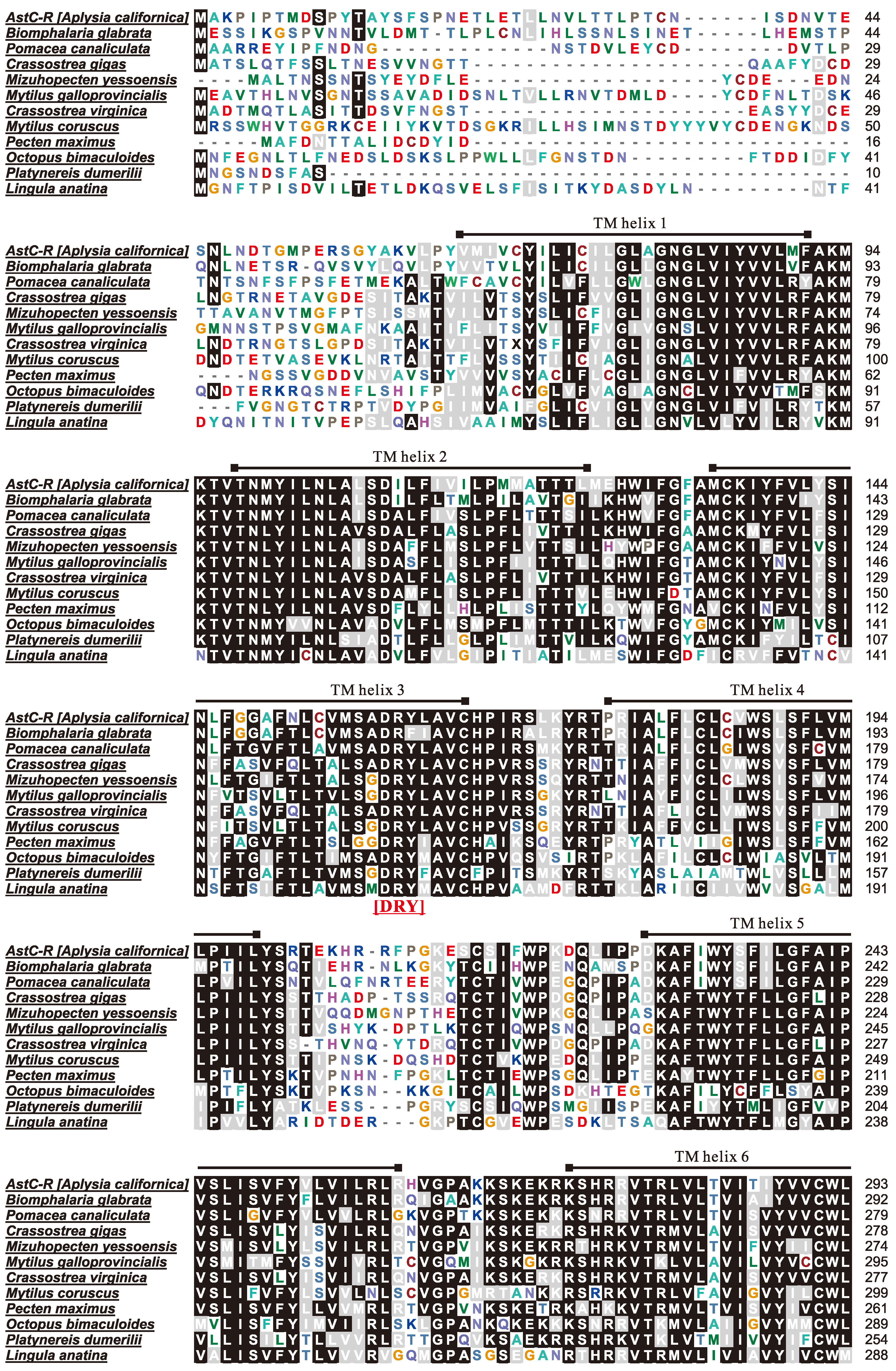


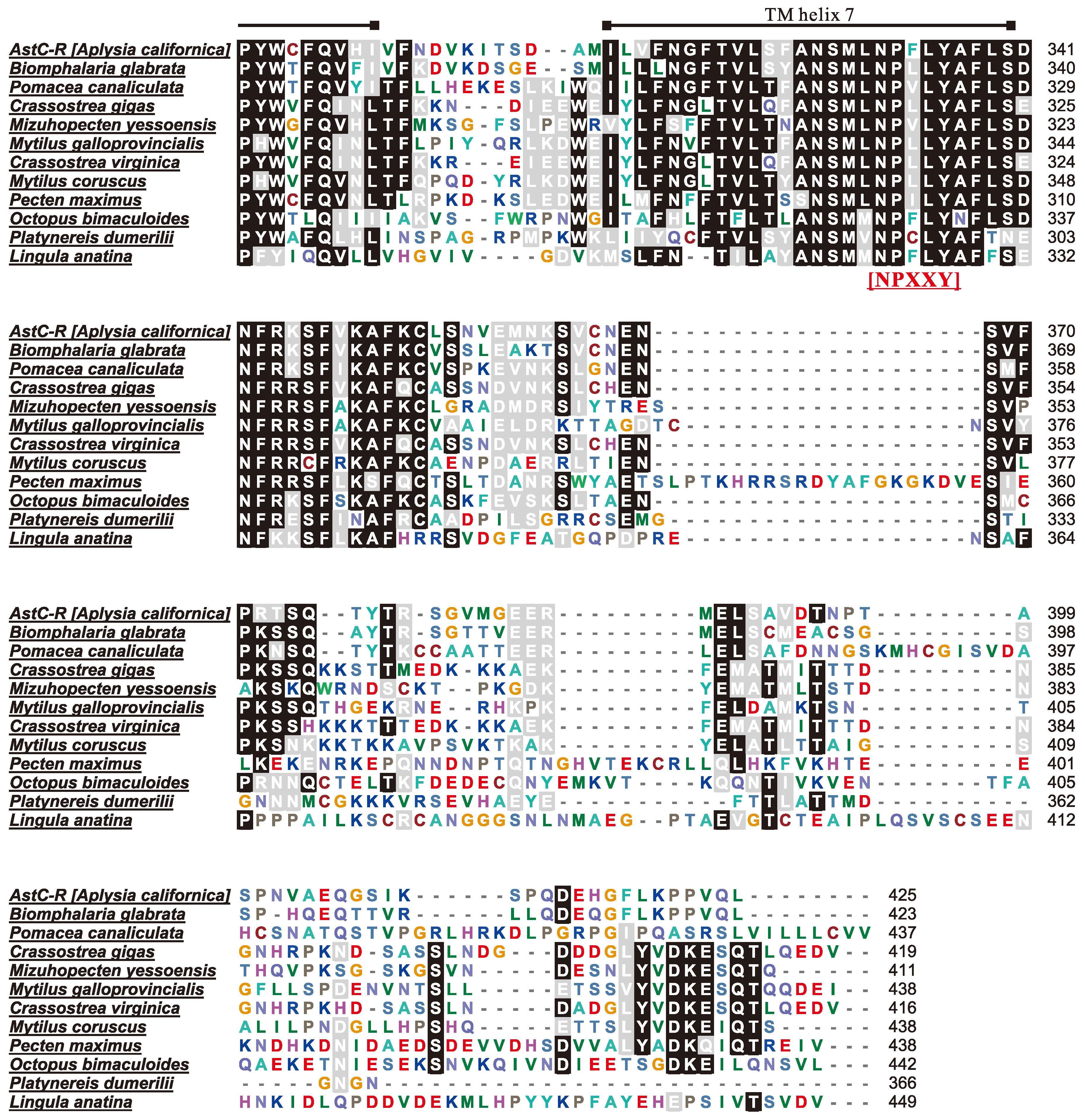


**Supplementary Figure 3. Comparison of AstC-R in *Aplysia* with sequences in other molluscs, an annelid (*Platynereis dumerilii*) and a Brachiopod (*Lingula anatine*).** The alignment is performed using BioEdit v5.0.6 (ClustalW Multiple alignment - Graphic View). Predicted 7 transmembrane (TM) helixes and the two shared motifs at TM helix 3 (DRY) and at TM helix 7 (NPXXY) are indicated.

**Supplementary Figure 4. Peptide synthesis and quality information from commercial companies**. See Supplementary Fig. 4.pdf.
