# Supplementary material for: Identification of an allatostatin C signaling system in mollusc Aplysia
Source: Sci Rep. 2022 Jan 24;12:1213. doi: 10.1038/s41598-022-05071-8 (PMC8786951; doi:10.1038/s41598-022-05071-8)
Supplement: Supplementary file 2 — Supplementary Figure 4. [file 41598_2022_5071_MOESM2_ESM.pdf]

# Contents

**AstC_COA** ........ **1**

AstC_HPLC ........ 2

AstC_LCMS........ 3

**AstC-*D*_COA** ........ **4**

AstC-*D*_HPLC........ 5

AstC-*D*_LCMS ........ 6

**AstC-*C*_COA** ........ **7**

AstC-*C*_HPLC ........ 8

AstC-*C*_LCMS ........ 9

**AstC-*L*_COA** ........ **10**

AstC-*L*_HPLC ........ 11

AstC-*L*_LCMS........ 12

**AstC-*T*_COA** ........ **13**

AstC-*T*_HPLC ........ 14

AstC-*T*_LCMS ........ 15

**AstC'_COA** ........ **16**

AstC'_HPLC ........ 17

AstC'_LCMS........ 18

**AstCG-DP_COA** ........ **19**

AstCG-DP_HPLC ........ 20

AstC-DP_LCMS ........ 21

**SPTR_COA** ........ **22**

SPTR_HPLC........ 23

SPTR_MS ........ 24

# • AstC_COA

## CERTIFICATE OF ANALYSIS

| | |
|---|---|
| **Product Name** | P21592-2 |
| **Catalog No.** | N/A |
| **Lot No.** | P21592-2-20201222 |
| **Sequence** | SHYSSMCMFNVVACY |
| **Length** | 15AA |
| **Modification** | C7-C14 |
| **Molecular Weight (MW)** | 1740.04 |
| **Storage** | -20°C |

| **Test Items** | **Specifications** | **Results** |
|---|---|---|
| **MW by MS** | 1740.0 | Conforms |
| **Purity by HPLC** | > 95% | 96.625% |
| **Peptide Content** | N/A | N/A |
| **Acetic acid content** | N/A | N/A |
| **Appearance** | White to off-white lyophilized powde | Conforms |
| **Quantity** | 2mg*5vials | 10mg |

Certified by: Melinda      Date 01/20/2021

Quality Assurance Department

# • AstC_HPLC

生工® Sangon Biotech

Sample Information

| | | |
|---|---|---|
| Name | : | P21592-2 |
| Sequence | : | SHYSSMCMFNVVACY |
| Modification | : | C7-C14 |
| Lot.No | : | P21592-2-20201222 |
| Pump A | : | 0.1%trifluoroacetic in 100%water |
| Pump B | : | 0.1%trifluoroacetic in 100%acetonrtrile |
| Total Flow | : | 1.0ml/min |
| Wavelength | : | 214nm |
| Analytical column type | : | SHIMADZU Inertsil ODS-SP(4.6*150MM*5UM) |
| Dissolution method | : | 0.5mg sample dissolved to 0.5mL by 100%H2O |
| Acquisition Time | : | 2021/01/20 08:21:08 |
| Inj.Volume | : | 60ul |

| Time | Module | Action | Value |
|---|---|---|---|
| 0.01 | Pumps | B.Conc | 28 |
| 20.00 | Pumps | B.Conc | 48 |

Chromatogram

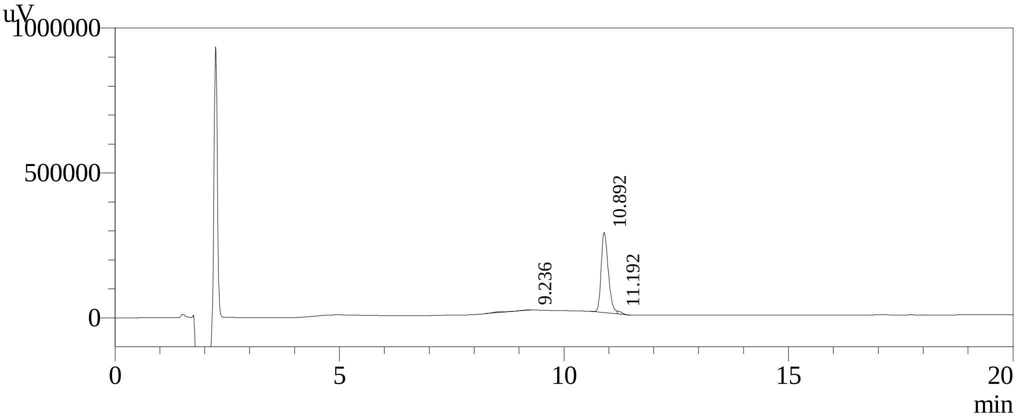


1 Det.A Ch1 / 214nm

PeakTable

Detector A Ch1 214nm

| Peak# | Ret. Time | Area | Height | Area % | Height % |
|---|---|---|---|---|---|
| 1 | 9.236 | 43689 | 186 | 1.376 | 0.065 |
| 2 | 10.892 | 3067778 | 276415 | 96.625 | 96.565 |
| 3 | 11.192 | 63462 | 9647 | 1.999 | 3.370 |
| Total | | 3174929 | 286248 | 100.000 | 100.000 |


**生工生物工程（上海）股份有限公司**

地址: 上海市松江区香闵路698号
电话/Tel: 400-821-0268
邮箱/Email: sales@sangon.com

Add: 698 Xiang Min Road SongJiang Shanghai China
传真/Fax: 86-21-37772170
网址/Web: www.sangon.com

## • AstC_LCMS

生工® Sangon Biotech

MS Spectrum

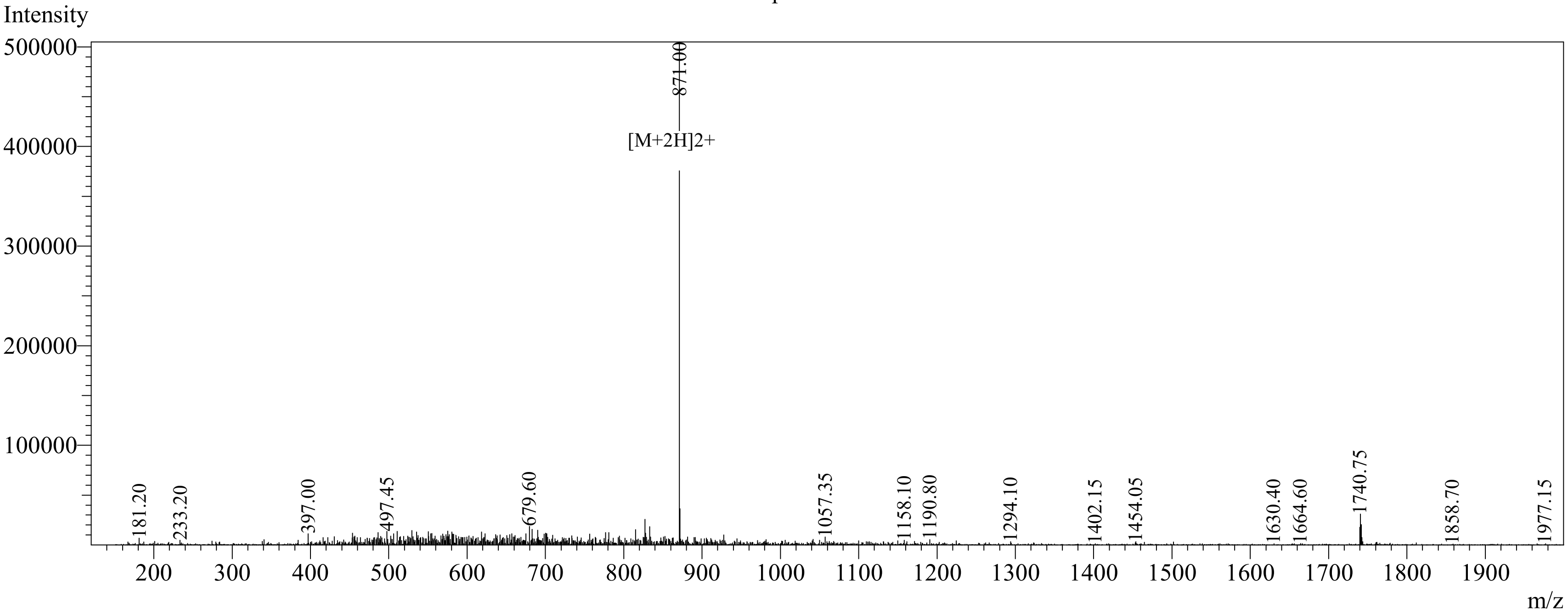


Sample Information
Dissolution method :0.1mg sample dissolved to 0.5mL by 100%H2O
Date Acquired :2021/01/19 09:28:14
Injection Volume :1ul

Interface :ESI
Nebulizing Gas Flow :1.50L/min
CDL Temp :250°C
CDL Volt :0v
Block Temp :200

Prerod Bias :+4.5kv
Detector :-0.2kv
T.Flow :0.2ml/min
B.conc :50%H2O/50%MeOH

Name :P21592-2
Sequence :SHYSSMCMFNVVACY
Modification :C7-C14
Lot No. :P21592-2-20201222
Theoretical :1740.041
bserved :1740.00

**生工生物工程（上海）股份有限公司**

地址：上海市松江区香闵路698号
电话/Tel: 400-821-0268
邮箱/Email: Sales@sangon.com

Add: 698 Xiang Min Road Songjiang Shanghai China
传真/Fax: 86-21-37772170
网址/Web: wwww.sangon.com

- # AstC-*D*_COA

## CERTIFICATE OF ANALYSIS

| | |
|---|---|
| **Product Name** | P23180-2(AST-C-D) |
| **Catalog No.** | N/A |
| **Lot No.** | P23180-2(AST-C-D)-20210707 |
| **Sequence** | TYSSMCMFNVVACY |
| **Length** | 14AA |
| **Modification** | C6-C13 |
| **Molecular Weight (MW)** | 1616.93 |
| **Storage** | -20°C |

| **Test Items** | **Specifications** | **Results** |
|---|---|---|
| **MW by MS** | 1616.8 | Conforms |
| **Purity by HPLC** | > 95% | 95.005% |
| **Peptide Content** | N/A | N/A |
| **Acetic acid content** | N/A | N/A |
| **Appearance** | White to off-white lyophilized powde | Conforms |
| **Quantity** | 1mg*10vials | 11.9mg |

Certified by: Melinda    Date 07/23/2021

Quality Assurance Department

# • AstC-*D*_HPLC

生工® Sangon Biotech

Sample Information

| | | |
|---|---|---|
| Name | : | P23180-2(AST-C-D) |
| Sequence | : | TYSSMCMFNVVACY |
| Modification | : | C6-C13 |
| Lot.No | : | P23180-2(AST-C-D)-20210707 |
| Pump A | : | 0.1%trifluoroacetic in 100%water |
| Pump B | : | 0.1%trifluoroacetic in 100%acetonrtrile |
| Total Flow | : | 1.0ml/min |
| Wavelength | : | 214nm |
| Analytical column type | : | SHIMADZU Inertsil ODS-SP(4.6*150MM*5UM) |
| Dissolution method | : | 0.5mg sample dissolved to 0.5mL by 5%HCOOH 25%ACN and 70%H2O |
| Acquisition Time | : | 2021/07/23 13:20:41 |
| Inj.Volume | : | 60ul |

| Time | Module | Action | Value |
|---|---|---|---|
| 0.01 | Pumps | B.Conc | 33 |
| 20.00 | Pumps | B.Conc | 53 |

Chromatogram

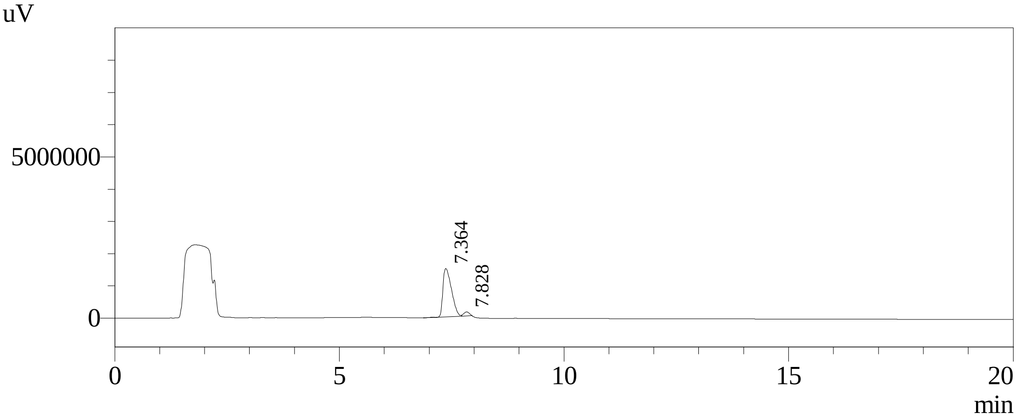


1 Det.A Ch1 / 214nm

PeakTable

Detector A Ch1 214nm

| Peak# | Ret. Time | Area | Height | Area % | Height % |
|---|---|---|---|---|---|
| 1 | 7.364 | 19808441 | 1501301 | 95.005 | 92.288 |
| 2 | 7.828 | 1041391 | 125451 | 4.995 | 7.712 |
| Total | | 20849833 | 1626752 | 100.000 | 100.000 |


**生工生物工程（上海）股份有限公司**

地址: 上海市松江区香闵路698号
电话/Tel: 400-821-0268
邮箱/Email: sales@sangon.com

Add: 698 Xiang Min Road SongJiang Shanghai China
传真/Fax: 86-21-37772170
网址/Web: www.sangon.com

- **AstC-*D*_LCMS**

生工® Sangon Biotech

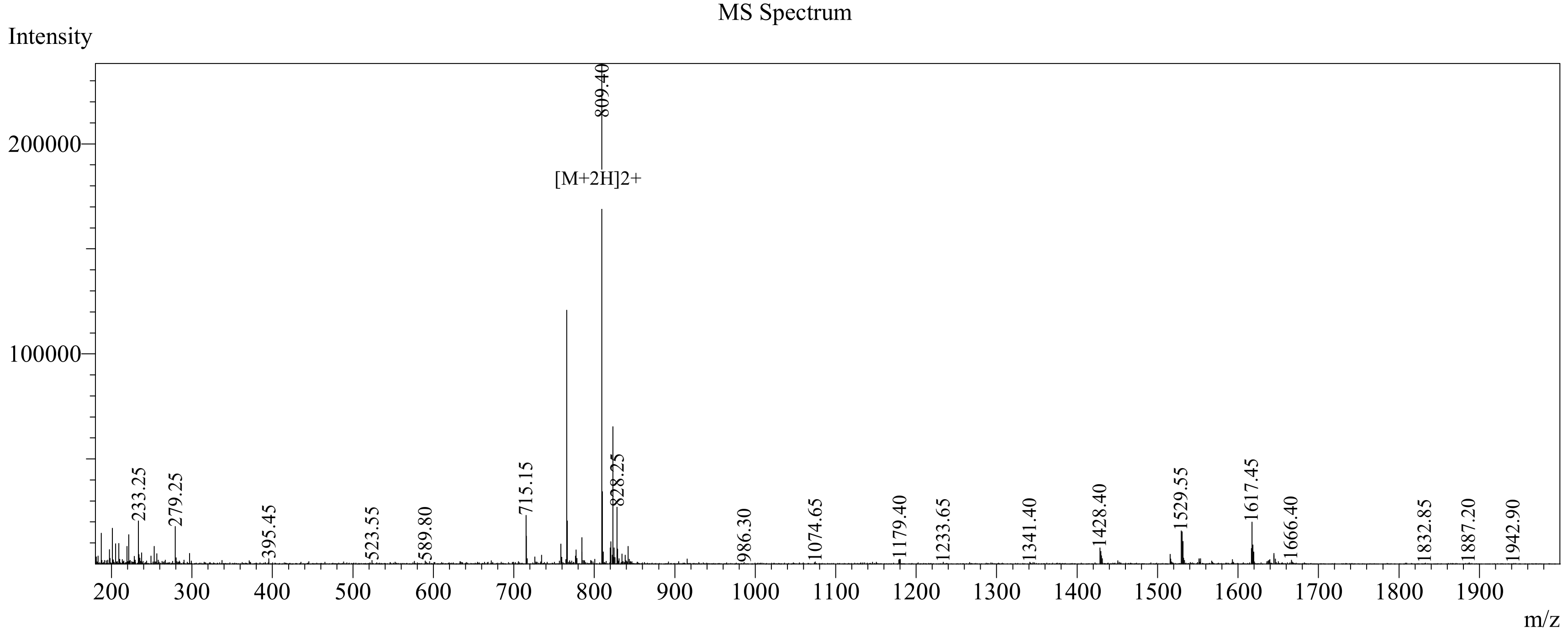


Sample Information
Dissolution method :0.1mg sample dissolved to 0.5mL by 5%HCOOH 25%ACN and 70%H2O
Date Acquired :2021/07/23 08:57:31
Injection Volume :1ul

Interface :ESI
Nebulizing Gas Flow :1.50L/min
CDL Temp :250°C
CDL Volt :0v
Block Temp :200

Prerod Bias :+4.5kv
Detector :-0.2kv
T.Flow :0.2ml/min
B.conc :50%H2O/50%MeOH

Name :P23180-2(AST-C-D)
Sequence :TYSSMCMFNVVACY
Modification :C6-C13
Lot No. :P23180-2(AST-C-D)-20210707
Theoretical :1616.93
bserved :1616.80

**生工生物工程（上海）股份有限公司**

地址：上海市松江区香闵路698号
Add: 698 Xiang Min Road Songjiang Shanghai China
电话/Tel: 400-821-0268
传真/Fax: 86-21-37772170
邮箱/Email: Sales@sangon.com
网址/Web: wwww.sangon.com

# • AstC-*C*_COA

## CERTIFICATE OF ANALYSIS

| | |
|---|---|
| **Product Name** | P23180-3(AST-C-C) |
| **Catalog No.** | N/A |
| **Lot No.** | P23180-3(AST-C-C)-20210707 |
| **Sequence** | SHIRCLVNVIACY |
| **Length** | 13AA |
| **Modification** | C5-C12 |
| **Molecular Weight (MW)** | 1488.80 |
| **Storage** | -20°C |

| **Test Items** | **Specifications** | **Results** |
|---|---|---|
| **MW by MS** | 1488.5 | Conforms |
| **Purity by HPLC** | > 95% | 95.549% |
| **Peptide Content** | N/A | N/A |
| **Acetic acid content** | N/A | N/A |
| **Appearance** | White to off-white lyophilized powde | Conforms |
| **Quantity** | 1mg*10vials | 15.0mg |

Certified by: Melinda    Date 07/23/2021

Quality Assurance Department

- **AstC_*C*_HPLC**

生工® Sangon Biotech

Sample Information

| | | |
|---|---|---|
| Name | : | P23180-3(AST-C-C) |
| Sequence | : | SHIRCLVNVIACY |
| Modification | : | C5-C12 |
| Lot.No | : | P23180-3(AST-C-C)-20210707 |
| Pump A | : | 0.1%trifluoroacetic in 100%water |
| Pump B | : | 0.1%trifluoroacetic in 100%acetonrtrile |
| Total Flow | : | 1.0ml/min |
| Wavelength | : | 214nm |
| Analytical column type | : | NanoChrom Chromcore TM120 C18(4.6*250MM*5UM) |
| Dissolution method | : | 0.5mg sample dissolved to 0.5mL by 100%H2O |
| Acquisition Time | : | 2021/07/23 09:11:37 |
| Inj.Volume | : | 40ul |

| Time | Module | Action | Value |
|---|---|---|---|
| 0.01 | Pumps | B.Conc | 29 |
| 20.00 | Pumps | B.Conc | 49 |

Chromatogram

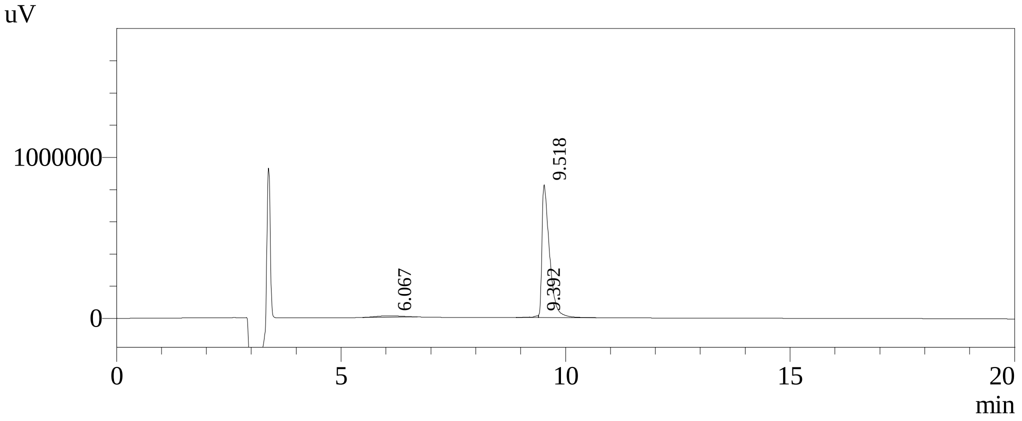


1 Det.A Ch1 / 214nm

PeakTable

Detector A Ch1 214nm

| Peak# | Ret. Time | Area | Height | Area % | Height % |
|---|---|---|---|---|---|
| 1 | 6.067 | 340378 | 8471 | 3.343 | 1.002 |
| 2 | 9.392 | 112732 | 11882 | 1.107 | 1.405 |
| 3 | 9.518 | 9727341 | 825057 | 95.549 | 97.593 |
| Total | | 10180451 | 845409 | 100.000 | 100.000 |

**生工生物工程（上海）股份有限公司**

地址: 上海市松江区香闵路698号
电话/Tel: 400-821-0268
邮箱/Email: sales@sangon.com

Add: 698 Xiang Min Road SongJiang Shanghai China
传真/Fax: 86-21-37772170
网址/Web: www.sangon.com

- **AstC_*C*_LCMS**

生工® Sangon Biotech

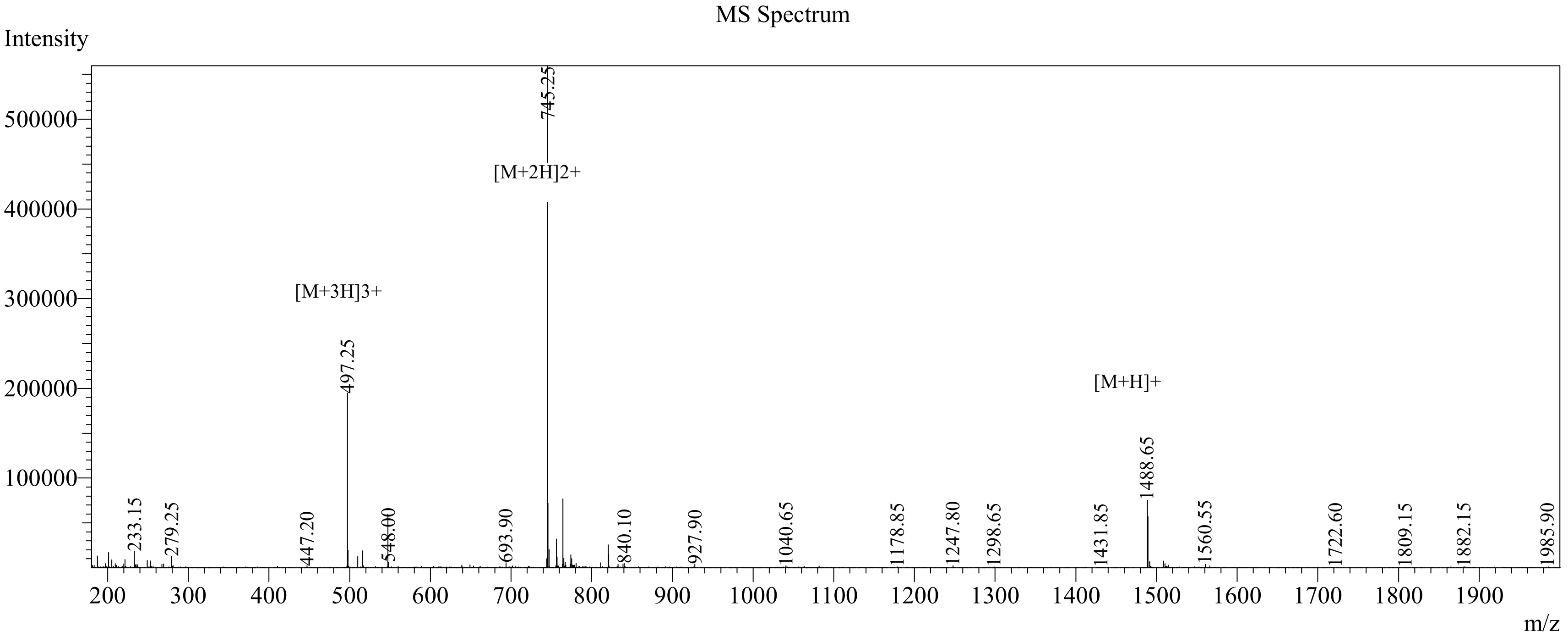


Sample Information
Dissolution method :0.1mg sample dissolved to 0.5mL by 100%H2O
Date Acquired :2021/07/23 08:55:18
Injection Volume :1ul

Interface :ESI
Nebulizing Gas Flow :1.50L/min
CDL Temp :250°C
CDL Volt :0v
Block Temp :200

Prerod Bias :+4.5kv
Detector :-0.2kv
T.Flow :0.2ml/min
B.conc :50%H2O/50%MeOH

Name :P23180-3(AST-C-C)
Sequence :SHIRCLVNVIACY
Modification :C5-C12
Lot No. :P23180-3(AST-C-C)-20210707
Theoretical :1488.80
bserved :1488.50

**生工生物工程（上海）股份有限公司**

地址：上海市松江区香闵路698号
Add: 698 Xiang Min Road Songjiang Shanghai China
电话/Tel: 400-821-0268
传真/Fax: 86-21-37772170
邮箱/Email: Sales@sangon.com
网址/Web: wwww.sangon.com

- **AstC-*L*_COA**

## CERTIFICATE OF ANALYSIS

| | |
|---|---|
| **Product Name** | P23180-4(AST-C-L) |
| **Catalog No.** | N/A |
| **Lot No.** | P23180-4(AST-C-L)-20210707 |
| **Sequence** | SHVMCLVNLISCF |
| **Length** | 13AA |
| **Modification** | C5-C12 |
| **Molecular Weight (MW)** | 1463.81 |
| **Storage** | -20°C |

| **Test Items** | **Specifications** | **Results** |
|---|---|---|
| **MW by MS** | 1463.6 | Conforms |
| **Purity by HPLC** | > 95% | 95.039% |
| **Peptide Content** | N/A | N/A |
| **Acetic acid content** | N/A | N/A |
| **Appearance** | White to off-white lyophilized powde | Conforms |
| **Quantity** | 1mg*10vials | 14.5mg |

Certified by: Melinda          Date 07/23/2021

Quality Assurance Department

- **AstC-*L*_HPLC**

生工® Sangon Biotech

Sample Information

| | | |
|---|---|---|
| Name | : | P23180-4(AST-C-L) |
| Sequence | : | SHVMCLVNLISCF |
| Modification | : | C5-C12 |
| Lot.No | : | P23180-4(AST-C-L)-20210707 |
| Pump A | : | 0.1%trifluoroacetic in 100%water |
| Pump B | : | 0.1%trifluoroacetic in 100%acetonrtrile |
| Total Flow | : | 1.0ml/min |
| Wavelength | : | 214nm |
| Analytical column type | : | NanoChrom Chromcore TM120 C18(4.6*250MM*5UM) |
| Dissolution method | : | 0.5mg sample dissolved to 0.5mL by 5%HCOOH 25%ACN and 70%H2O |
| Acquisition Time | : | 2021/07/23 12:30:48 |
| Inj.Volume | : | 40ul |

| Time | Module | Action | Value |
|---|---|---|---|
| 0.01 | Pumps | B.Conc | 38 |
| 20.00 | Pumps | B.Conc | 58 |

Chromatogram

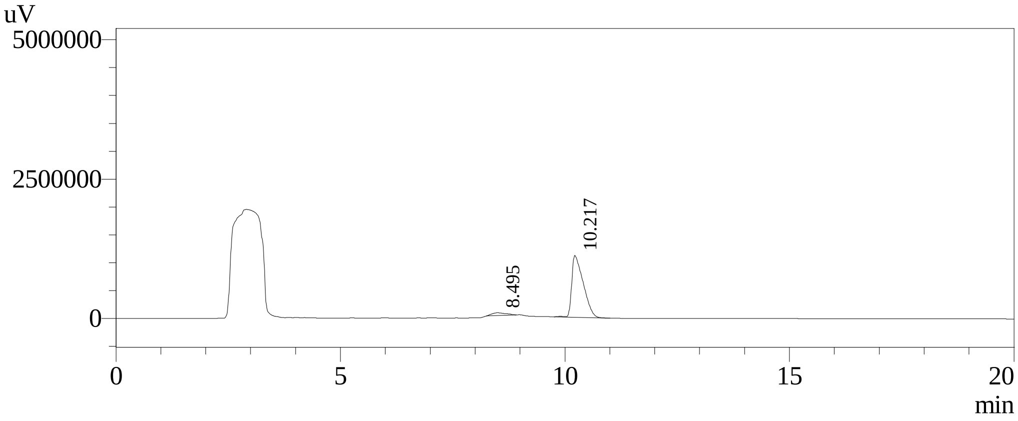


1 Det.A Ch1 / 214nm

PeakTable

Detector A Ch1 214nm

| Peak# | Ret. Time | Area | Height | Area % | Height % |
|---|---|---|---|---|---|
| 1 | 8.495 | 1030958 | 48086 | 4.961 | 4.158 |
| 2 | 10.217 | 19750780 | 1108358 | 95.039 | 95.842 |
| Total | | 20781739 | 1156444 | 100.000 | 100.000 |


**生工生物工程（上海）股份有限公司**

地址: 上海市松江区香闵路698号 Add: 698 Xiang Min Road SongJiang Shanghai China
电话/Tel: 400-821-0268 传真/Fax: 86-21-37772170
邮箱/Email: sales@sangon.com 网址/Web: www.sangon.com

- **AstC-*L*_LCMS**

生工® Sangon Biotech

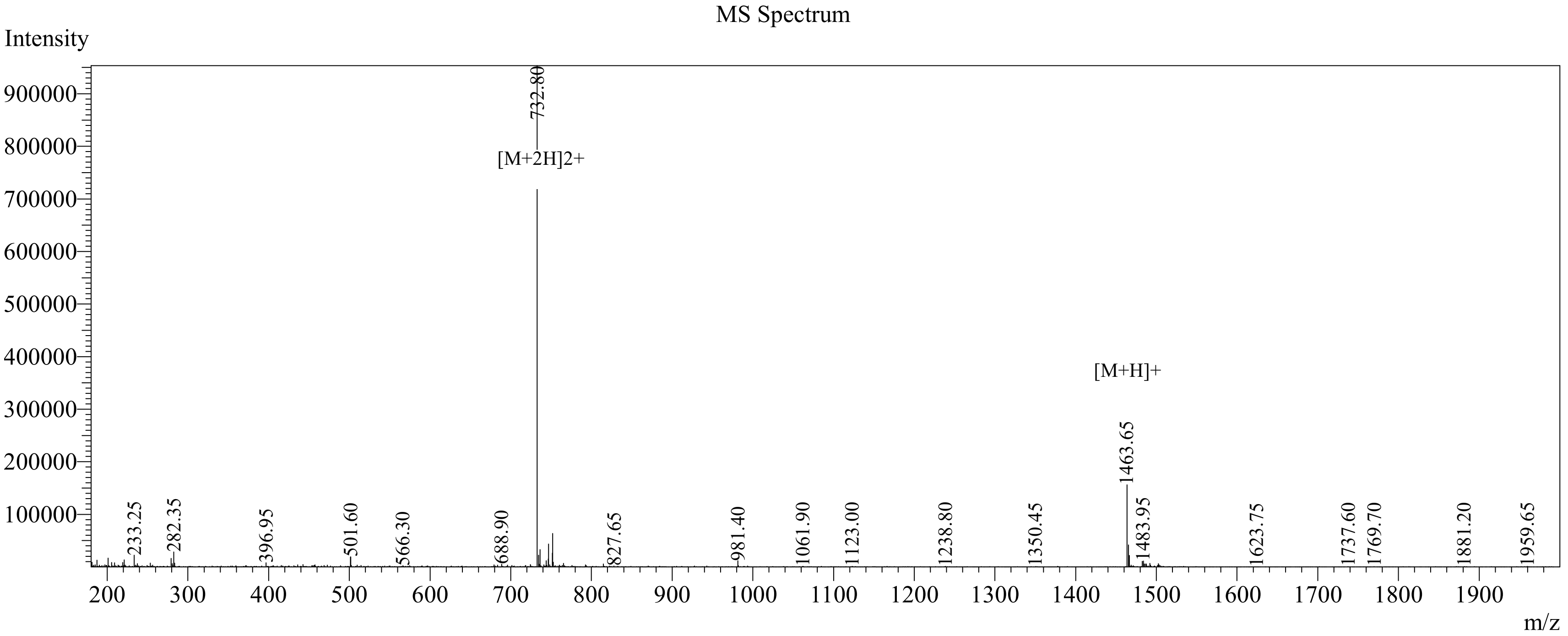


Sample Information
Dissolution method :0.1mg sample dissolved to 0.5mL by 100%H2O
Date Acquired :2021/07/23 12:34:28
Injection Volume :1ul

Interface :ESI
Nebulizing Gas Flow :1.50L/min
CDL Temp :250°C
CDL Volt :0v
Block Temp :200

Prerod Bias :+4.5kv
Detector :-0.2kv
T.Flow :0.2ml/min
B.conc :50%H2O/50%MeOH

Name :P23180-4(AST-C-L)
Sequence :SHVMCLVNLISCF
Modification :C5-C12
Lot No. :P23180-4(AST-C-L)-20210707
Theoretical :1463.81
bserved :1463.60


**生工生物工程（上海）股份有限公司**

地址：上海市松江区香闵路698号 Add: 698 Xiang Min Road Songjiang Shanghai China
电话/Tel: 400-821-0268 传真/Fax: 86-21-37772170
邮箱/Email: Sales@sangon.com 网址/Web: wwww.sangon.com

- **AstC-*T*_COA**

## CERTIFICATE OF ANALYSIS

| | |
|---|---|
| **Product Name** | P23180-5(AST-C-T) |
| **Catalog No.** | N/A |
| **Lot No.** | P23180-5(AST-C-T)-20210707 |
| **Sequence** | SHYNSMCMFNVVACY |
| **Length** | 15AA |
| **Modification** | C7-C14 |
| **Molecular Weight (MW)** | 1767.07 |
| **Storage** | -20°C |

| **Test Items** | **Specifications** | **Results** |
|---|---|---|
| **MW by MS** | 1766.8 | Conforms |
| **Purity by HPLC** | > 95% | 95.308% |
| **Peptide Content** | N/A | N/A |
| **Acetic acid content** | N/A | N/A |
| **Appearance** | White to off-white lyophilized powde | Conforms |
| **Quantity** | 1mg*10vials | 14.0mg |

Certified by: Melinda  Date 07/23/2021

Quality Assurance Department

• **AstC-*T*_HPLC**

生工® Sangon Biotech

Sample Information

| | | |
|---|---|---|
| Name | : | P23180-5(AST-C-T) |
| Sequence | : | SHYNSMCMFNVVACY |
| Modification | : | C7-C14 |
| Lot.No | : | P23180-5(AST-C-T)-20210707 |
| Pump A | : | 0.1%trifluoroacetic in 100%water |
| Pump B | : | 0.1%trifluoroacetic in 100%acetonrtrile |
| Total Flow | : | 1.0ml/min |
| Wavelength | : | 214nm |
| Analytical column type | : | SHIMADZU Inertsil ODS-SP(4.6*150MM*5UM) |
| Dissolution method | : | 0.5mg sample dissolved to 0.5mL by 100%H2O |
| Acquisition Time | : | 2021/07/23 12:50:50 |
| Inj.Volume | : | 60ul |

| Time | Module | Action | Value |
|---|---|---|---|
| 0.01 | Pumps | B.Conc | 30 |
| 20.00 | Pumps | B.Conc | 50 |

Chromatogram

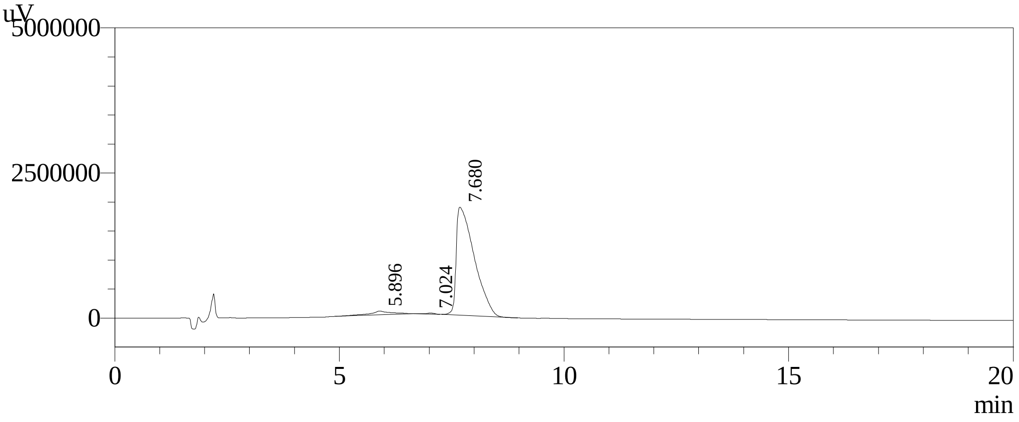


1 Det.A Ch1 / 214nm

PeakTable

Detector A Ch1 214nm

| Peak# | Ret. Time | Area | Height | Area % | Height % |
|---|---|---|---|---|---|
| 1 | 5.896 | 2279032 | 67010 | 4.157 | 3.439 |
| 2 | 7.024 | 293463 | 19995 | 0.535 | 1.026 |
| 3 | 7.680 | 52250427 | 1861291 | 95.308 | 95.534 |
| Total | | 54822923 | 1948295 | 100.000 | 100.000 |


生工生物工程（上海）股份有限公司

地址: 上海市松江区香闵路698号
电话/Tel: 400-821-0268
邮箱/Email: sales@sangon.com

Add: 698 Xiang Min Road SongJiang Shanghai China
传真/Fax: 86-21-37772170
网址/Web: www.sangon.com

- # AstC-*T*_LCMS

生工® Sangon Biotech

MS Spectrum

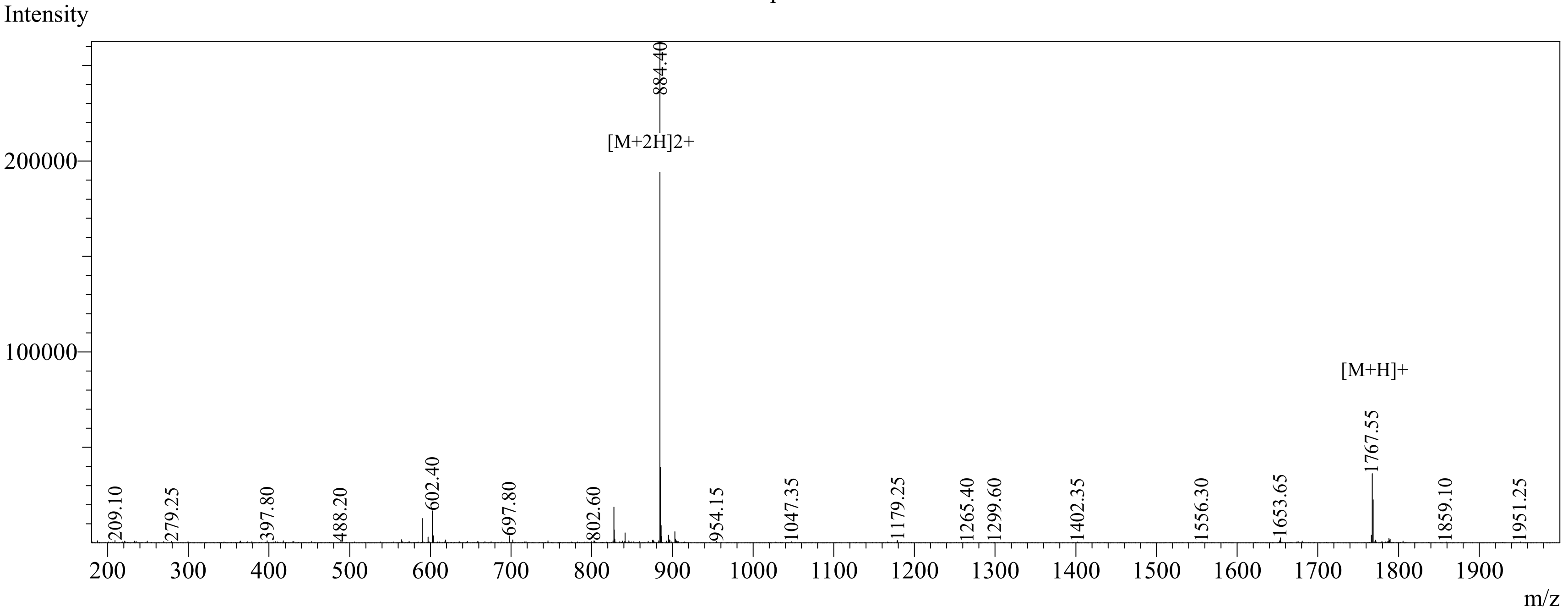


Sample Information
Dissolution method :0.1mg sample dissolved to 0.5mL by 100%H2O
Date Acquired :2021/07/23 13:00:38
Injection Volume :1ul

Interface :ESI
Nebulizing Gas Flow :1.50L/min
CDL Temp :250°C
CDL Volt :0v
Block Temp :200

Prerod Bias :+4.5kv
Detector :-0.2kv
T.Flow :0.2ml/min
B.conc :50%H2O/50%MeOH

Name :P23180-5(AST-C-T)
Sequence :SHYNSMCMFNVVACY
Modification :C7-C14
Lot No. :P23180-5(AST-C-T)-20210707
Theoretical :1767.07
bserved :1766.80


**生工生物工程（上海）股份有限公司**

地址：上海市松江区香闵路698号 Add: 698 Xiang Min Road Songjiang Shanghai China
电话/Tel: 400-821-0268 传真/Fax: 86-21-37772170
邮箱/Email: Sales@sangon.com 网址/Web: wwww.sangon.com

# • AstC'_COA



## CERTIFICATE OF ANALYSIS

| | |
|---|---|
| **Order ID** | GP120461-1 |
| **Name** | N/A |
| **Lot No.** | GP120461-1-0707 |
| **Sequence** | SHYSSMCMFNVVACY |
| **Dissolution condition** | 5%HAC+15%ACN+80%H2O |
| **Length** | 15AA |
| **Modification** | N/A |
| **Molecular Weight (MW)** | 1742.03 |
| **Storage** | -20°C |

| **Test Items** | **Specifications** | **Results** |
|---|---|---|
| **MW by MS** | 1741.40 | Conforms |
| **Purity by HPLC** | >95% | 95.046% |
| **Peptide Content** | N/A | N/A |
| **Moisture content** | N/A | N/A |
| **Acetic acid content** | N/A | N/A |
| **Appearance** | White to off-white lyophilized powder | Conforms |
| **Quantity** | 10mg | 1.0mg*10 |

Certified by: LiuHui　　Date 07/28/2021

Quality Assurance Department

**Note: this product is intended for research use only; not for diagnostic or human use.**

Guoping Pharmaceutical Co., LTD

地址:合肥市经开区桃花工业园拓展区工投立恒工业广场A2西F1,电话:0551-62841987 传真:0551-62841765 www.guopingyaoye.com

# Sample Information

Order ID :GP120461-1
Name :N/A
Sequence :SHYSSMCMFNVVACY
Lot.No :GP120461-1-0707
Pump A :0.1%Trifluoroacetic in 100% water
Pump B :0.1%Trifluoroacetic in 100% acetonitrile
Total Flow :1ml/min
Wavelength :220nm
Analytical column type :SHIMADZU Inertsil ODS-SP(4.6*250mm*5um)
Dissolution method :5%HAC+15%ACN+80%H2O
Inj. Volume :19 uL

| Time | Module | Action | Value |
|---|---|---|---|
| 0.01 | Pumps | B.Conc | 15 |
| 20.00 | Pumps | B.Conc | 55 |
| 30.00 | Pumps | B.Conc | 100 |
| 38.00 | Pumps | B.Conc | 100 |
| 40.00 | Pumps | B.Conc | 15 |
| 50.00 | Controller | Stop | |

# Chromatogram

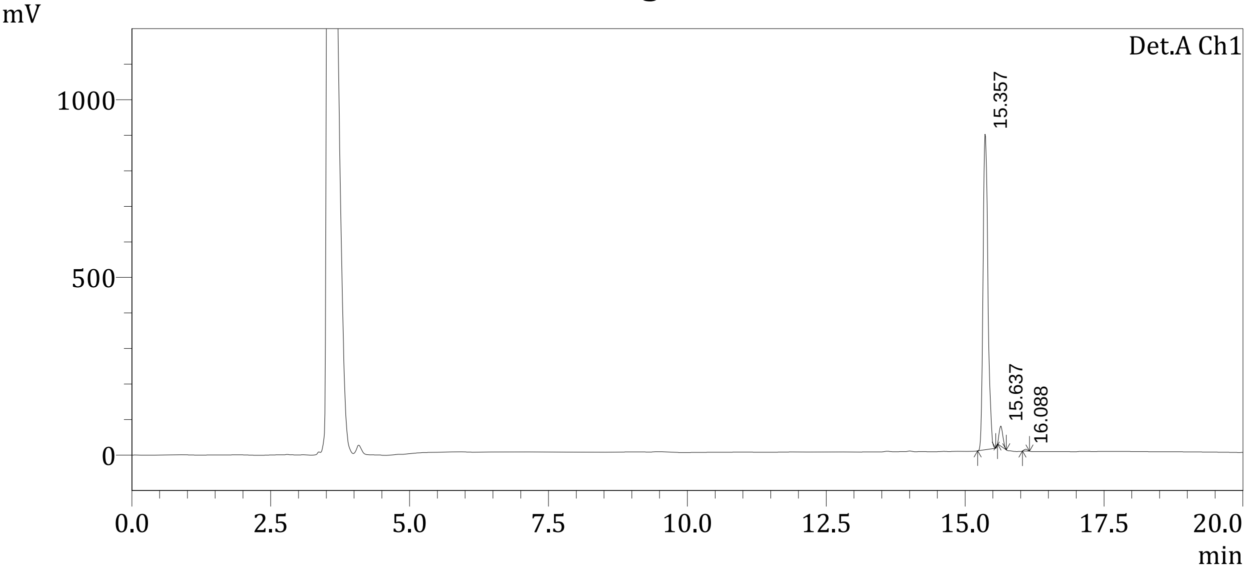


1 Det.A Ch1/220nm

PeakTable

Detector A Ch1 220nm

| Peak# | Ret. Time | Area | Height | Area % | Height % |
|---|---|---|---|---|---|
| 1 | 15.357 | 5374298 | 888305 | 95.046 | 93.552 |
| 2 | 15.637 | 259024 | 56302 | 4.581 | 5.929 |
| 3 | 16.088 | 21091 | 4926 | 0.373 | 0.519 |
| Total | | 5654412 | 949532 | 100.000 | 100.000 |

- **AstC'_LCMS**

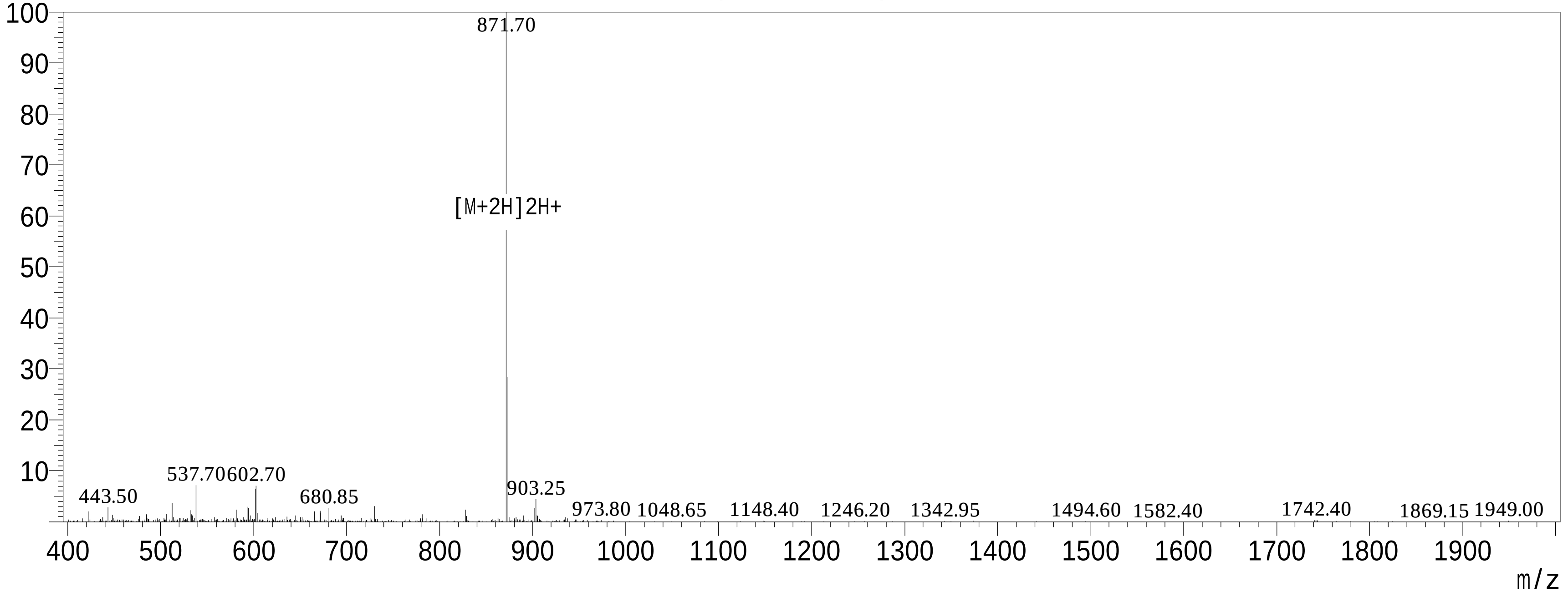


Sample Information

| | | | |
|---|---|---|---|
| Dissolution method | :5%HAC+8%ACN+87%H2O | Interface | :ESI |
| Modified Date | :2021/07/27 | Nebulizing Gas Flow | :1.50L/min |
| Injection Volume | :1ul | CDL Temp | :250C |
| Heat Block Temp | :200 | CDL Volt | :0v |
| Prerod Bias | :+1.5kv | | |
| Detector | :-0.2kv | | |
| T.Flow | :0.2ml/min | | |
| B.conc | :50%H2O/50%MEOH | | |

| | |
|---|---|
| Order ID | :GP120461-1 |
| Name | :N/A |
| Sequence | :SHYSSMCMFNVVACY |
| Lot.No | :GP120461-1-0707 |
| Theoretical | :1742.03 |
| Observed | :1741.40 |

- # AstCG-DP_COA

安徽省国平药业有限公司

## CERTIFICATE OF ANALYSIS

| | |
|---|---|
| **Order ID** | GP120340-3 |
| **Name** | N/A |
| **Lot No.** | GP120340-3-1110 |
| **CAS** | AVIPVSSPEPMEEASALQLLPGKI -NH2 |
| **Dissolution condition** | 100%H2O |
| **Length** | 24AA |
| **Modification** | N/A |
| **Molecular Weight (MW)** | 2475.88 |
| **Storage** | -20°C |

| **Test Items** | **Specifications** | **Results** |
|---|---|---|
| **MW by MS** | 2474.90 | Conforms |
| **Purity by HPLC** | >95% | 95.083% |
| **Peptide Content** | N/A | N/A |
| **Moisture content** | N/A | N/A |
| **Acetic acid content** | N/A | N/A |
| **Appearance** | White to off-white lyophilized powder | Conforms |
| **Quantity** | 10mg | 2.0mg*5 |

Certified by: LiuHui Date 12/08/2020

Quality Assurance Department

**Note: this product is intended for research use only; not for diagnostic or human use.**

Guoping Pharmaceutical Co., LTD

地址:合肥市经开区桃花工业园拓展区工投立恒工业广场 A2西F1,电话:0551-62841987 传真:0551-62841765 www.guopingyaoye.com

## Sample Information

Order ID :GP120340-3
Name :N/A
Sequence :AVIPVSSPEPMEEASALQLLPGKI-NH2
Lot.No :GP120340-3-1110
Pump A :0.1%Trifluoroacetic in 100% water
Pump B :0.1%Trifluoroacetic in 100% acetonitrile
Total Flow :1ml/min
Wavelength :220nm
Analytical column type :SHIMADZU Inertsil ODS-SP(4.6*250mm*5um)
Dissolution method :100%H2O
Inj. Volume :11 uL

| Time | Module | Action | Value |
|---|---|---|---|
| 0.01 | Pumps | B.Conc | 10 |
| 20.00 | Pumps | B.Conc | 50 |
| 30.00 | Pumps | B.Conc | 100 |
| 38.00 | Pumps | B.Conc | 100 |
| 40.00 | Pumps | B.Conc | 10 |
| 50.00 | Controller | Stop | |

## Chromatogram

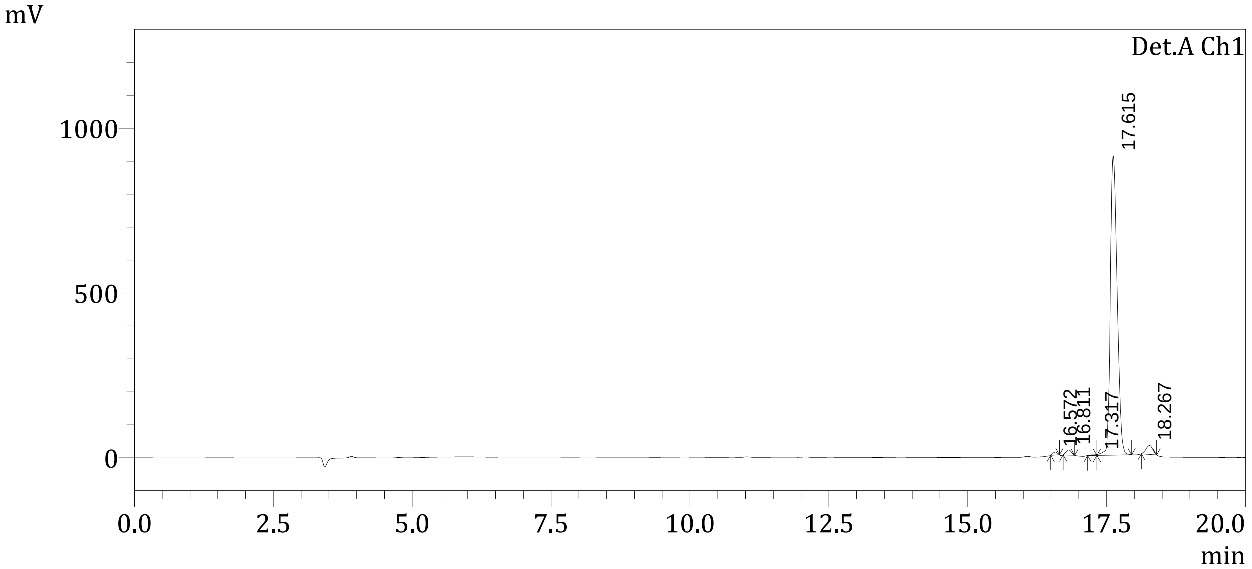


1 Det.A Ch1/220nm

PeakTable

Detector A Ch1 220nm

| Peak# | Ret. Time | Area | Height | Area % | Height % |
|---|---|---|---|---|---|
| 1 | 16.572 | 49447 | 8963 | 0.607 | 0.931 |
| 2 | 16.811 | 105710 | 15594 | 1.297 | 1.620 |
| 3 | 17.317 | 17298 | 2019 | 0.212 | 0.210 |
| 4 | 17.615 | 7751737 | 908548 | 95.083 | 94.408 |
| 5 | 18.267 | 228387 | 27235 | 2.801 | 2.830 |
| Total | | 8152578 | 962359 | 100.000 | 100.000 |

GUOPING PHARMACEUTICAL

# • AstCG-DP_MS

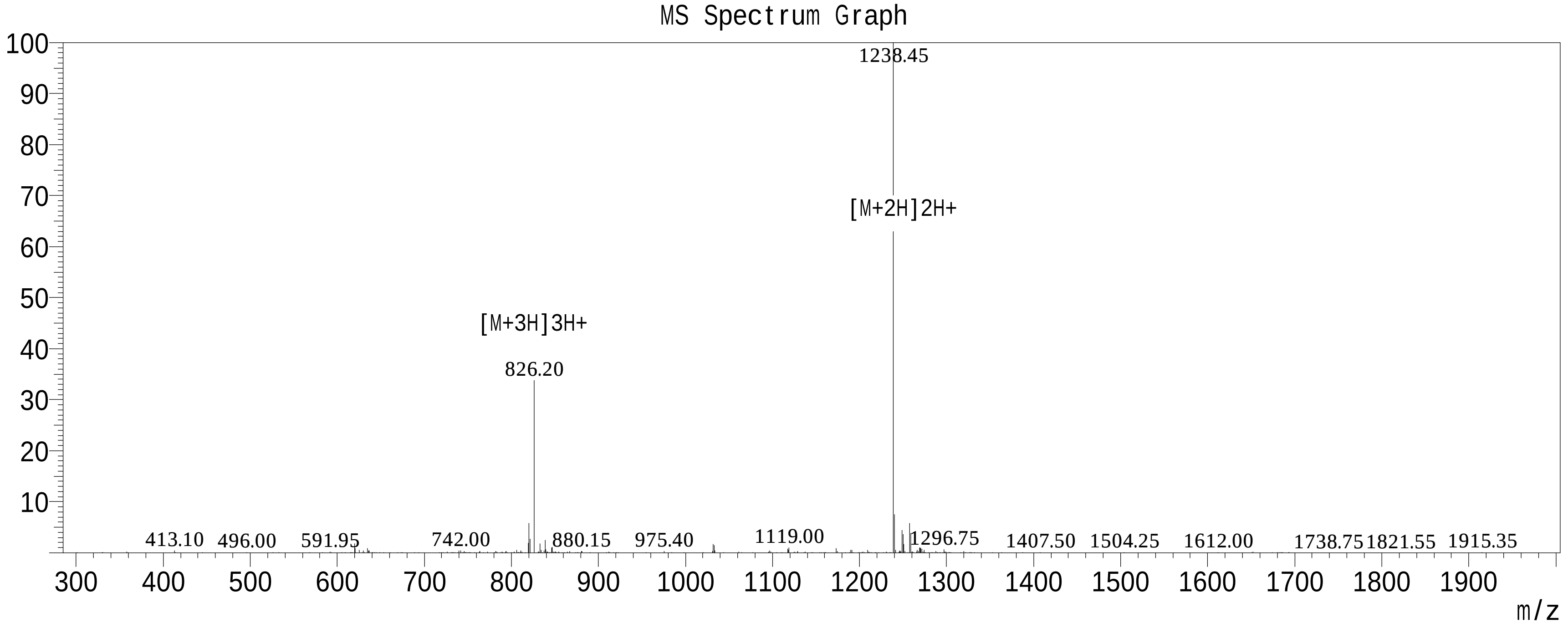


## Sample Information

| | | | | | |
|---|---|---|---|---|---|
| Dissolution method | :5%HAC+8%ACN+87%H2O | Interface | :ESI | Prerod Bias | :+1.5kv |
| Modified Date | :2020/12/07 | Nebulizing Gas Flow | :1.50L/min | Detector | :-0.2kv |
| Injection Volume | :1ul | CDL Temp | :250C | T.Flow | :0.2ml/min |
| Heat Block Temp | :200 | CDL Volt | :0v | B.conc | :50%H2O/50%MEOH |

| | |
|---|---|
| Order ID | :GP120340-3 |
| Name | :N/A |
| Sequence | :AVIPVSSPEPMEEASALQLLPGKI-NH2 |
| Lot.No | :GP120340-3-1110 |
| Theoretical | :2475.88 |
| Observed | :2474.90 |

ChinaPeptides Co., Ltd.

Tel(Shanghai): +86-21-50795728
Tel(Suzhou): +86-512-63930660
E-mail: inside@chinapeptides.com
outside@chinapeptides.com
Http://www.chinapeptides.com

# Certificate of Analysis

**Product Name:** 04010030919

**Sequence:** MRYMGICMKKQYNNFIPFPCLRS-NH2 (Remark:Disulfide bond)

**Sequence(Three Letters Code):** Met-Arg-Tyr-Met-Gly-Ile-Cys-Met-Lys-Lys-Gln -Tyr-Asn-Asn-Phe-Ile-Pro-Phe-Pro-Cys-Leu-Arg-Ser-NH2 (Remark:Disulfide bond)

**Molecular Weight:** 2838.52

**HPLC Analysis:** Peptide Purity:98.39%

(See attached RP-HPLC chromatogram)

**MS Analysis:** ESI_MS

(See attached MS spectrum)

**Solubility:** 1mg/ml in 20%ACN/80%$H_2O$

(Please choose proper dissolution conditions according to your assay)

**Appearance:** lyophilized powder

**Counter Ion:** Trifluoroacetate

**Remarks:**

**For research purpose only.**

**Important: Storage conditions:-20° C,seal, avoid light,dry.**

**Quality Assurance By:** 陆介宇 **Date:** 03-21-2017

上海强耀生物科技有限公司 质检专用章



Innovation Professional Gratitude Win-win

Tel(Shanghai): +86-21-50795728
Tel(Suzhou): +86-512-63930660
E-mail: inside@chinapeptides.com
outside@chinapeptides.com
Http://www.chinapeptides.com

# HPLC Analysis Report

| | | | |
|---|---|---|---|
| Measurement: | Peak Area | Run Time: | 13min |
| Calculation Type: | Percent | Wavelength : | 220nm |
| Flow Rate : | 1ml/min | Inj.Vol: | 10uL |
| Buffer A : | 0.1% TFA in water | Buffer B: | 0.1%TFA in Acetonitrile |
| Column: | Kromasil 100-5C18,4.6mmX250mm,5 micron | Column Temp: | 25℃ |
| Gradient(linear): | 25%-45% buffer B in 13min | | |

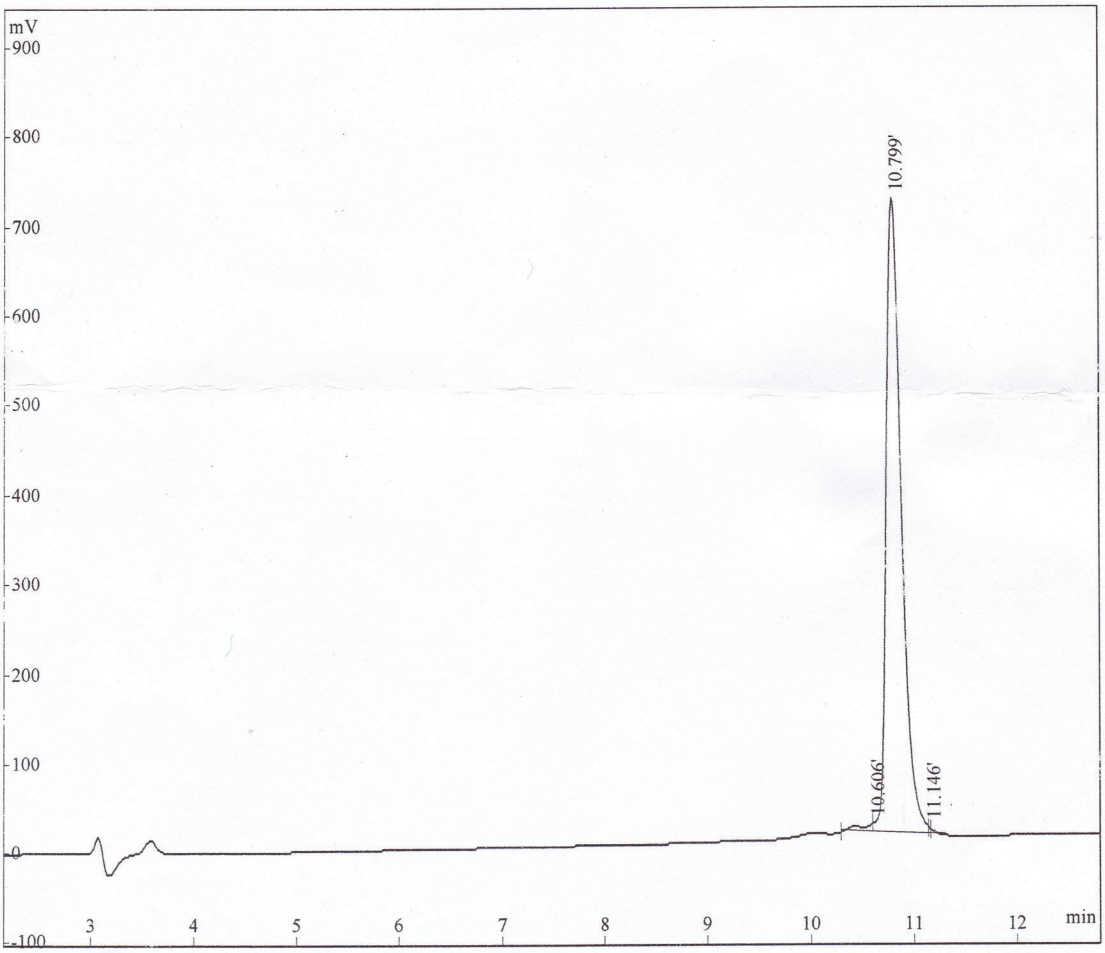


| Rank | Time | Name | Conc. | Area |
|---|---|---|---|---|
| 1 | 10.606 | | 1.476 | 108181 |
| 2 | 10.799 | | 98.39 | 7211386 |
| 3 | 11.146 | | 0.1328 | 9733 |
| Total | | | 100 | 7329300 |

Tel(Shanghai): +86-21-50795728
Tel(Suzhou): +86-512-63930660
E-mail: inside@chinapeptides.com
outside@chinapeptides.com
Http://www.chinapeptides.com

# MS Analysis Report

Expected MS: 2838.52

Mass Spectrometer: API 150EX

Ion Source: ESI

B.conc: 80%ACN/20%$H_2O$

NEB:10.00 CUR:12.00

IS:+4500 TEM:0.00

Flow Rate : 0.2ml/min

Run Time: 1min

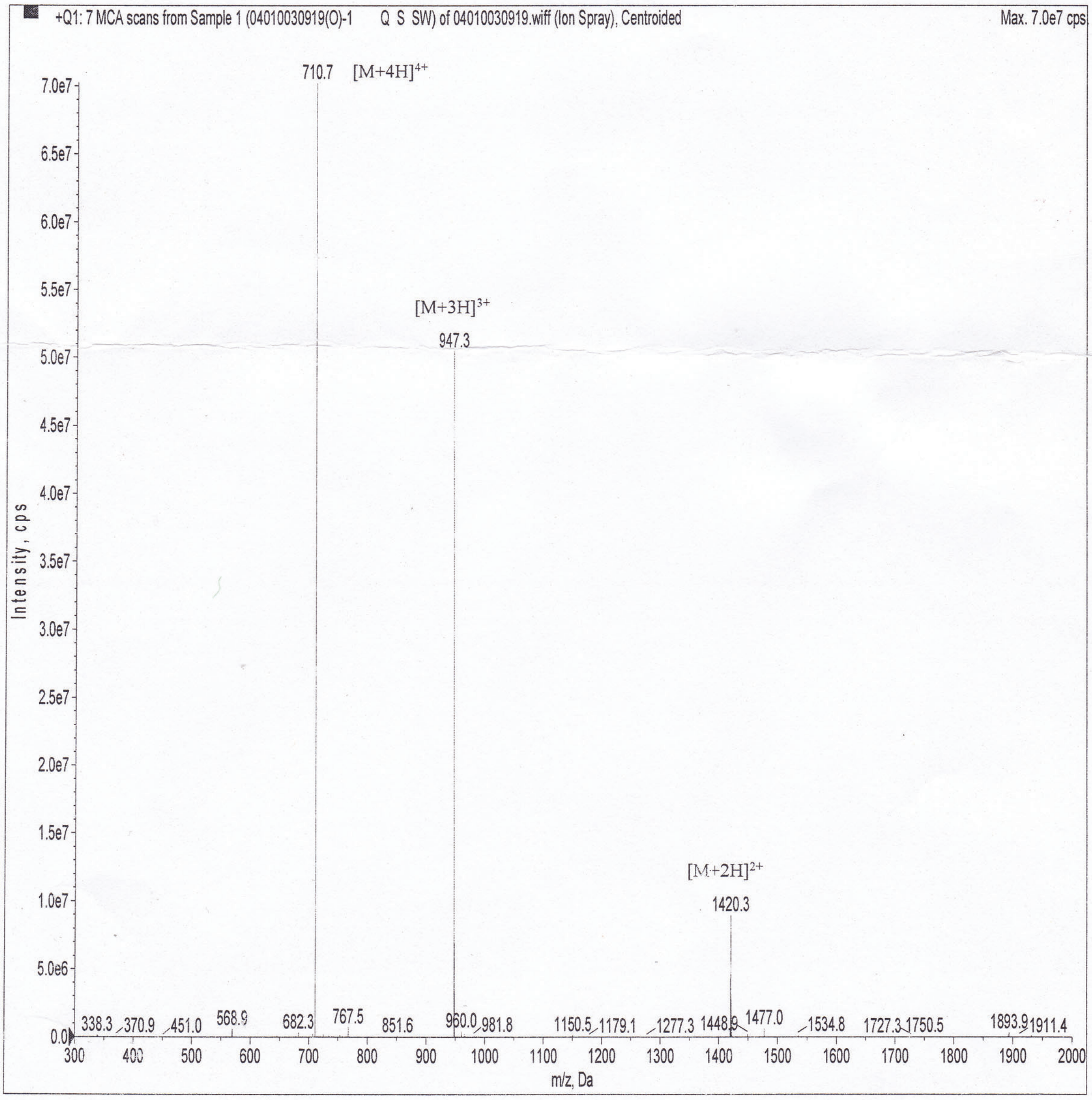




Innovation Professional Gratitude Win-win
